# The ubiquitin E3 ligase ARIH1 regulates hnRNP E1 protein stability, EMT and breast cancer progression

**DOI:** 10.1038/s41388-022-02199-9

**Published:** 2022-01-31

**Authors:** Breege V. Howley, Bidyut Mohanty, Annamarie Dalton, Simon Grelet, Joseph Karam, Toros Dincman, Philip H. Howe

**Affiliations:** 1 Department of Biochemistry and Molecular Biology, Medical University of South Carolina, Charleston, SC USA; 2 Department of Biochemistry and Molecular Biology, Mitchell Cancer Institute, University of South Alabama, Mobile, AL USA; 3 Department of Medicine, Medical University of South Carolina, Charleston, SC USA; 4 Hollings Cancer Center, Medical University of South Carolina, Charleston, SC USA

**Keywords:** Breast cancer, Metastasis

## Abstract

The epithelial to mesenchymal transition (EMT), a process that is aberrantly activated in cancer and facilitates metastasis to distant organs, requires coordinated transcriptional and post-transcriptional control of gene expression. The tumor-suppressive RNA binding protein, hnRNP-E1, regulates splicing and translation of EMT-associated transcripts and it is thought that it plays a major role in the control of epithelial cell plasticity during cancer progression. We have utilized yeast 2 hybrid screening to identify novel hnRNP-E1 interactors that play a role in regulating hnRNP-E1; this approach led to the identification of the E3 ubiquitin ligase ARIH1. Here, we demonstrate that hnRNP-E1 protein stability is increased upon ARIH1 silencing, whereas, overexpression of ARIH1 leads to a reduction in hnRNP-E1. Reduced ubiquitination of hnRNP-E1 detected in ARIH1 knockdown (KD) cells compared to control suggests a role for ARIH1 in hnRNP-E1 degradation. The identification of hnRNP-E1 as a candidate substrate of ARIH1 led to the characterization of a novel function for this ubiquitin ligase in EMT induction and cancer progression. We demonstrate a delayed induction of EMT and reduced invasion in mammary epithelial cells silenced for ARIH1. Conversely, ARIH1 overexpression promoted EMT induction and invasion. ARIH1 silencing in breast cancer cells significantly attenuated cancer cell stemness in vitro and tumor formation in vivo. Finally, we utilized miniTurboID proximity labeling to identify novel ARIH1 interactors that may contribute to ARIH1’s function in EMT induction and cancer progression.

## Introduction

The epithelial to mesenchymal transition normally occurs during embryonic development, fibrosis and wound healing, and can be aberrantly activated during cancer progression [1, 2]. The mesenchymal phenotype, induced during this transition, is characterized by a loss of cell-cell contacts and apical-basal polarity as well as the reorganization of cytoskeletal networks [3]. In cancer, EMT is associated with increased cell migration and invasion, and is linked to the cancer stem cell phenotype, which is thought to occur through the reactivation of embryonic self-renewal signaling pathways. Cancer stem cells (CSCs) may drive not only metastatic growth but also facilitate cancer recurrence and chemo- and radio-resistance [4].

The tumor suppressor hnRNP E1 (PCBP1) is an RNA binding protein that regulates diverse processes including translation and splicing [5]. We have shown that hnRNP E1 silences translation, in a TGFβ-dependent manner, by binding to C-rich elements in the 3′-UTR of select mRNAs [6–8]. Furthermore, hnRNP E1 regulates splicing of EMT-associated genes including CD44 and PNUTS [9, 10]. In normal murine mammary gland epithelial (NMuMG) cells, hnRNP E1 silencing increases migration and invasiveness in vitro and the formation of distant metastases in vivo [7]. In addition, hnRNP E1 knockdown (E1KD) cells acquire stem cell-like properties as assessed by mammosphere formation, stemness marker expression, and the ability to reconstitute cleared mammary fat pads in vivo [11].

ARIH1 is an E3 ubiquitin ligase that associates with and is activated by neddylated Cullin-RING ubiquitin ligase (CRL) complexes [12]. ARIH1 has been shown to transfer the first ubiquitin to CRL substrates, such as Cyclin E and Sec31A [13]. A role for ARIH1 in the ISGylation of proteins, with the ubiquitin-like modification ISG15, has also been described [14]. This atypical E3 ligase plays a role in several processes including mitophagy, myonuclear organization and DNA damage-induced translational arrest in embryonic stem cells and cancer cells [14–17]. The identification of hnRNP E1 as a candidate substrate of this ubiquitin ligase is an exciting area of research that may define a novel function for ARIH1 in EMT induction and cancer progression. Indeed, here we present data that suggests that ARIH1 acts as a key player in cancer progression.

## Results

### hnRNP E1 is a candidate substrate for ARIH1

**Fig. 1:**
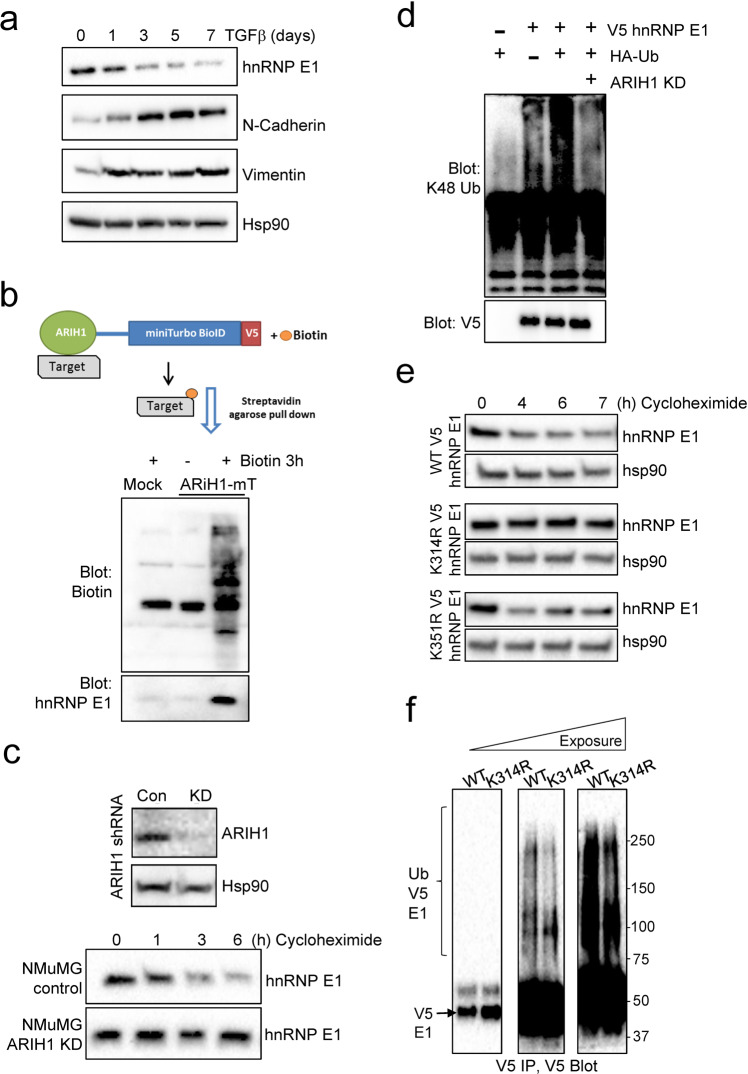
*hnRNP E1 is a candidate substrate for ARIH1*. **a** Expression of hnRNP E1 and the mesenchymal markers, N-cadherin and Vimentin, in NMuMG cells treated with TGFβ. Hsp90 was used as a loading control. **b** Schematic of ARIH1-miniTurboID pulldown strategy (top panel). Immunoblot of biotin and hnRNP E1 in Hek293 cells either mock transfected or transfected with ARIH1-miniTurboID and treated with or without Biotin for 3 h. **c** Stable KD of ARIH1 by shRNA (top panel) and hnRNP E1 protein stability in control and ARIH1 KD NMuMG cells (bottom panel). **d** Ubiquitination of exogenous V5 tagged hnRNP E1 detected by V5 immunoprecipitation and K48 ubiquitin immunoblot. **e** Protein stability, assessed by cycloheximide chase assay, of WT and K314R or K351R mutant V5-tagged hnRNP E1. **f** Ubiquitination of WT and K314R V5 tagged hnRNP E1 detected by V5 immunoprecipitation and immunoblot.

Previous research has demonstrated that hnRNP E1 plays an important role in EMT induction and tumor progression in breast cancer. hnRNP E1 mRNA is ubiquitously expressed across multiple cell types, however, little is known about how this RNA binding protein is regulated post-transcriptionally. We observe reduced hnRNP E1 protein levels upon long-term TGFβ treatment of NMuMG mammary epithelial cells, upregulation of N-cadherin and Vimentin indicated a TGFβ-induced EMT in this cell line (Fig. 1a). Under these conditions we do not observe a decrease in hnRNP E1 transcript levels (Supplementary Fig. S1a). Furthermore, polysome profiling experiments revealed no alteration in hnRNP E1 translation, suggesting the potential regulation of hnRNP E1 protein stability (Supplementary Fig. S1b). Consistent with this hypothesis, cycloheximide treatment significantly reduced hnRNP E1 protein levels in NMuMG cells, and this effect can be rescued by the proteasome inhibitor MG132 (Supplementary Fig. S1c).

We utilized yeast 2 hybrid screening to characterize novel hnRNP E1 interactors that may play a role in regulating hnRNP E1 expression (Supplementary Fig. S1d). This approach identified known interactors of hnRNP E1 including other hnRNP proteins (hnRNP LL) and splicing factors (SRSF3), in addition to several novel candidates including the E3 ubiquitin ligase ARIH1 (HHARI). ARIH1 was characterized further in our model due to its role in regulating protein stability via ubiquitination. To validate the interaction between ARIH1 and hnRNP E1, we performed miniTurboID proximity labeling using a promiscuous biotin ligase fused to ARIH1. This approach is particularly useful for transient interactions between E3 ligases and their substrates. Hek293 cells were transiently transfected with an ARIH1 miniTurboID construct, and 100 μM Biotin was added to cells 3 h prior to harvesting and streptavidin agarose pulldown of biotinylated proteins. hnRNP E1 immunoblotting revealed pulldown of hnRNP E1 in the biotin-treated ARIH1 miniTurboID transfected lysates but not in mock transfected or no biotin control samples (Fig. 1b).

In order to test the role of ARIH1 in regulating hnRNP E1 protein stability, we stably silenced ARIH1 in NMuMG cells. In this model, we observe increased hnRNP E1 protein stability upon ARIH1 knockdown (KD), when cells are treated with cycloheximide to block protein synthesis (Fig. 1c). Increased hnRNP E1 protein levels were also observed using siRNA to transiently silence ARIH1 in this cell line (Supplementary Fig. S1e). Consistent with these findings, hnRNP E1 protein levels increase following treatment with the neddylation inhibitor MLN4924, which inhibits CRL-mediated activation of ARIH1. (Supplementary Fig. S1f). Next, we tested the effect of ARIH1 silencing on V5-tagged hnRNP E1 ubiquitination. A reduction in hnRNP E1 K48 ubiquitination was observed in ARIH1 knockdown cells, compared to control, indicating a role for ARIH1 in the ubiquitination and subsequent degradation of hnRNP E1 (Fig. 1d, Supplementary Fig. S1g). To further characterize the effect of ubiquitination on hnRNP E1 protein stability, we utilized the PhosphoSitePlus [18] database to identify lysine residues in hnRNP E1 that are modified by ubiquitin across multiple curated studies; this search identified K314 and K351 as the most commonly ubiquitin modified residues. To test their contribution to hnRNP E1 protein stability in our model, we generated lysine-to-arginine (K-to-R) mutants of V5-tagged hnRNP E1 and looked at protein stability by cycloheximide chase assay. We observed increased protein stability of K314R hnRNP E1, and to a lesser extent K351R, when compared to WT protein (Fig. 1e, Supplementary Fig. S1h). To strengthen these findings, we tested WT and K314R V5-tagged hnRNP E1 ubiquitination and observed a reduction in ubiquitination levels when compared to WT protein (Fig. 1f). Overall, these data indicate that ubiquitination of K314 is an important regulator of hnRNP E1 protein stability.

### ARIH1 is upregulated during TGFβ induced EMT and modulates EMT induction

**Fig. 2:**
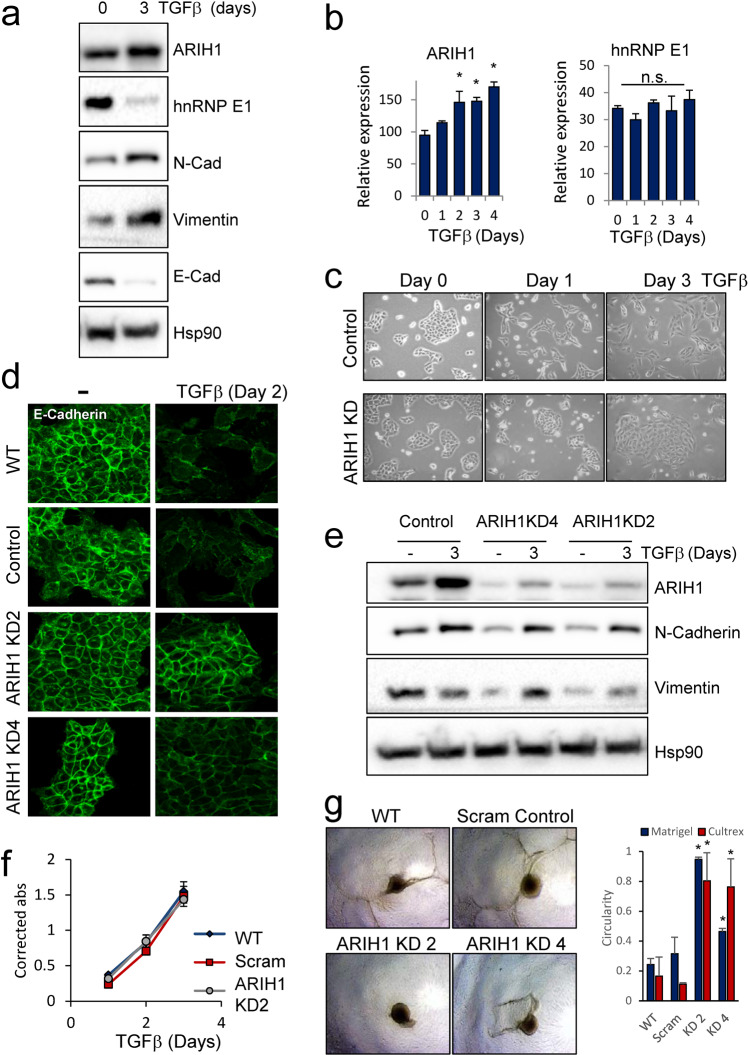
*ARIH1 silencing delays EMT in mammary epithelial cells*. **a** Protein levels of hnRNP E1, ARIH1, epithelial marker E-cadherin and the mesenchymal markers N-Cadherin and Vimentin in TGFβ-treated NMuMG cells. **b** *ARIH1* and *hnRNP E1* mRNA levels following TGFβ treatment of NMuMG cells as assessed by RNA seq analysis of duplicate time points from GSE114572. (Mean ± SD, one-way ANOVA with Bonferroni post-hoc test; *P < 0.05, n.s Not significant). **c** Cell morphology of NMuMG control and ARIH1 KD cells following TGFβ treatment. **d** Expression of the epithelial marker E-cadherin in NMuMG control and ARIH1 KD cells following 2 d of TGFβ treatment. **e** Induction of the mesenchymal marker N-Cadherin and Vimentin in NMuMG control and ARIH1 KD cells following 3 d of TGFβ treatment. **f** Proliferation of NMuMG control and ARIH1 KD cells as assessed by MTT assay. **g** 3D invasion assay of TGFβ treated NMuMG control and ARIH1 KD cells; representative images of Cultrex invasion assay (left panel) and quantitation of spheroid circularity (Mean ± SD, paired *t*-test compared to WT; *P < 0.05).

As hnRNP E1 protein levels are decreased upon long-term TGFβ treatment, we next assessed ARIH1 protein expression under these conditions. An increase in ARIH1 protein by 3 d of TGFβ treatment in NMuMG cells was detected by immunoblot (Fig. 2a). Analysis of RNA seq data from GSE114572 using untreated and TGFβ treated NMuMG cells demonstrated a significant increase in ARIH1 transcript (Fig. 2b). No change in hnRNP E1 transcript expression was observed under these conditions, consistent with a post-transcription mechanism of hnRNP E1 regulation. Following this observation, we assessed the effect of ARIH1 silencing on EMT induction in mammary epithelial cells and observed a delayed EMT in cells silenced for ARIH1 and treated with TGFβ. An elongated mesenchymal cell morphology and increased expression of the mesenchymal marker N-Cadherin, loss of the epithelial marker E-cadherin, and actin cytoskeletal reorganization were observed in control NMuMG cells at day 1–3 of TGFβ treatment (Fig. 2c–e, Supplementary Fig. S2a, b). In contrast, ARIH1 KD cells remained in an epithelial state during this time frame. As the transition to a mesenchymal phenotype is associated with increased invasiveness linked to cancer progression, we assessed cell invasion in our model. Initially, we determined the cell proliferation rate of WT, scrambled control, and ARIH1 KD cells, with no significant difference in proliferation observed between these cell lines (Fig. 2f). 3D invasion assays were performed using cells grown in spheroids and embedded in a basement membrane mimic (Matrigel or Cultrex). Cells were pretreated with TGFβ for 3 d, and we observed reduced invasion of ARIH1 KD cells, as compared to WT and scrambled control cells (Fig. 2g, Supplementary Fig. S2c). The circularity of spheroids was used as a measure of invasiveness, with invasive spheroids demonstrating a reduced circularity index (<0.6), compared to round, non-invasive spheroids with values close to 1 (Fig. 2g).

**Fig. 3:**
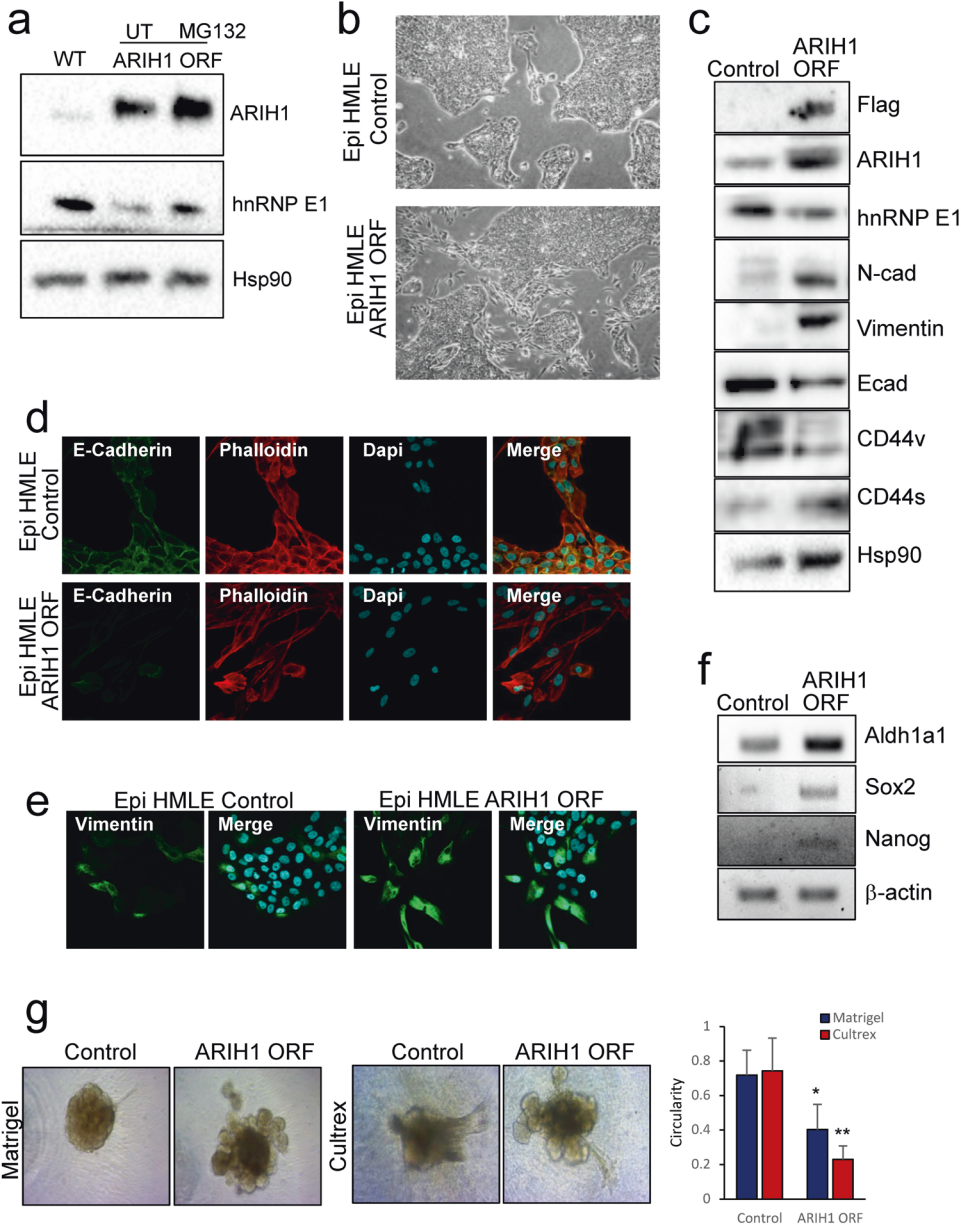
*ARIH1 overexpression promotes epithelial cell plasticity*. **a** Immunoblot of hnRNP E1, ARIH1, and Hsp90 (loading control) following ARIH1 overexpression in a HMLE epithelial subpopulation (HMLE Epi). **b** Cell morphology of HMLE Epi control and ARIH1 ORF cells. **c** Protein levels of hnRNP E1, the epithelial marker E-cadherin, the mesenchymal markers N-Cadherin and Vimentin, and CD44v and CD44s isoforms in HMLE Epi control and ARIH1 ORF cells. **d** E-cadherin and phalloidin immunofluorescence of HMLE Epi control and ARIH1 ORF cells. **e** Vimentin immunofluorescence of HMLE Epi control and ARIH1 ORF cells. **f** mRNA expression of the cancer stemness markers *ALDH1A1, SOX2* and *NANOG* assessed by semi-quantitative PCR, *BACTIN* was used as a loading control. **g** 3D invasion assay of HMLE Epi control and ARIH1 ORF cells; representative images of Matrigel and Cultrex invasion assays (left panel) and quantitation of spheroid circularity (Mean ± SD, unpaired *t*-test compared to control; *P < 0.05, **P < 0.01).

In order to test the effect of ARIH1 overexpression on hnRNP E1 protein expression and EMT induction, we used an epithelial subpopulation (CD24^high^, CD44^low^) of the human mammary HMLE cell line. Human ARIH1 ORF was overexpressed in this line, resulting in reduced hnRNP E1 protein levels, this reduction was partially rescued by treatment with the proteasome inhibitor MG132 (Fig. 3a). Next, we assessed EMT induction in cells expressing empty vector or ARIH1 ORF. Stable expression of ARIH1 in this cell line increased cell plasticity with a transition of border cells to a mesenchymal phenotype observed by microscopy (Fig. 3b). Increased expression of the mesenchymal markers N-cadherin, Vimentin and CD44s and decreased epithelial markers, E-Cadherin and CD44v, were observed by immunoblot (Fig. 3c). Consistent with these findings, decreased E-Cadherin, increased Vimentin and actin cytoskeleton reorganization was observed in transitioned border cells by immunofluorescence (Fig. 3d, e). An increase in the cancer stem cell markers *ALDH1A1, SOX2*, and *NANOG* were also observed by PCR (Fig. 3f). Finally, increased invasion was observed in cell overexpressing ARIH1 as determined by 3D invasion assay (Fig. 3g).

### ARIH1 modulates cancer cell invasion, stemness and tumor progression

**Fig. 4:**
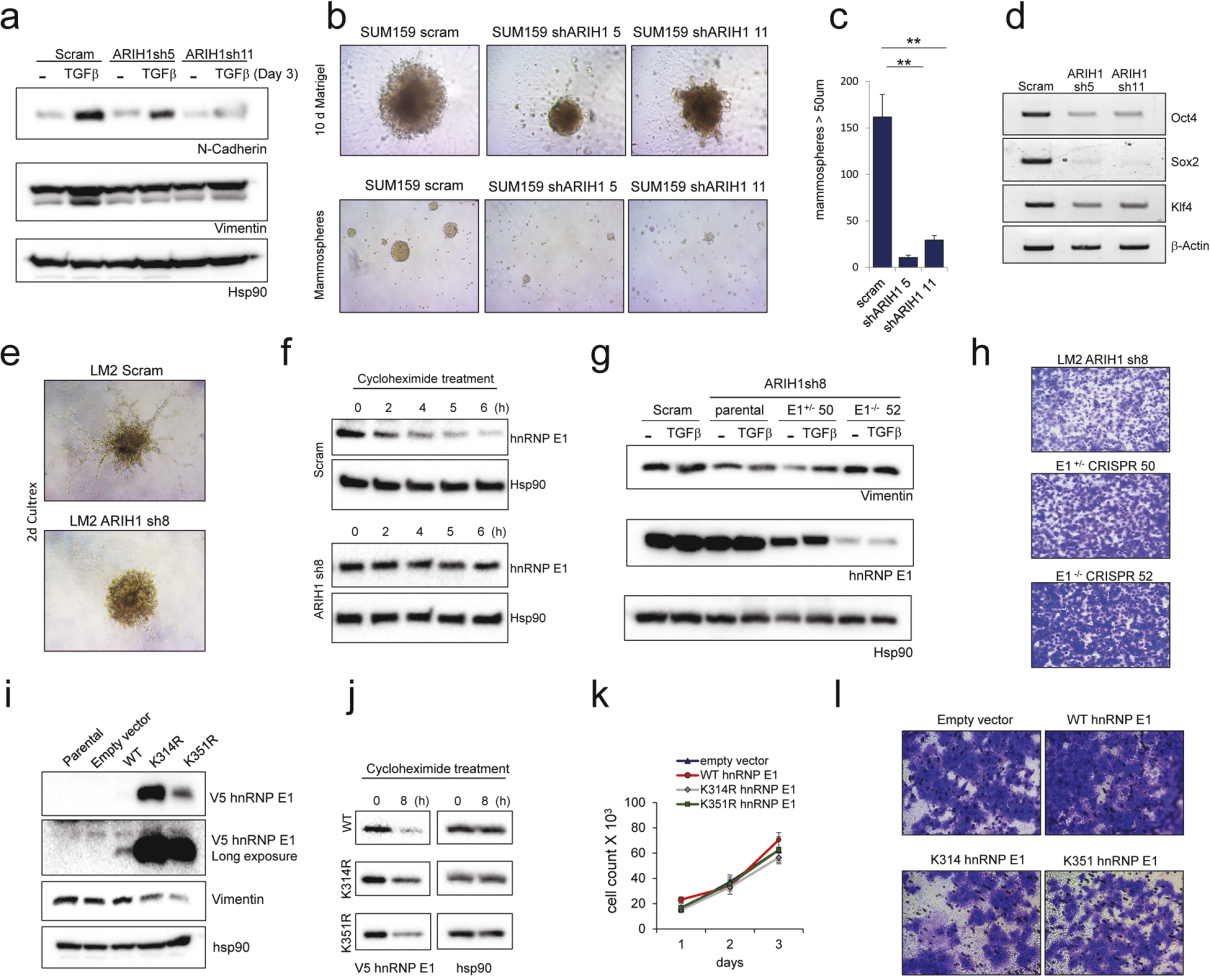
*ARIH1 regulates cancer cell invasion and stemness*. **a** Protein levels of the mesenchymal markers N-Cadherin and Vimentin in TGFβ-treated SUM159 scrambled control and ARIH1 KD cells. **b** Representative images of SUM159 scrambled control and ARIH1 KD cell invasion in Matrigel (top panel) and representative images of mammospheres (bottom panel). **c** Quantitation of sphere number (Mean ± SEM, paired *t*-test compared to control; **P < 0.01). **d** Transcript expression of the cancer stemness markers *POU5F1* (OCT4), *SOX2* and *KLF4* assessed by semi-quantitative PCR, *BACTIN* was used as a loading control. **e** Representative images of LM2 scrambled control and ARIH1 KD cell invasion in Cultrex. **f** Protein levels of hnRNP E1 in cycloheximide-treated LM2 scrambled control and ARIH1 KD cells. Hsp90 is used as a loading control. **g** Protein levels of the mesenchymal marker Vimentin in TGFβ-treated LM2 scrambled control, ARIH1 KD cells and ARIH1 KD with hnRNP E1 silencing. **h** Representative images of 2D invasion assay with LM2 ARIH1 KD cells and ARIH1 KD with hnRNP E1 silencing. **i** V5 hnRNP E1 and Vimentin protein levels in LM2 cells stably expressing WT, K314R and K351R V5 hnRNP E1. **j** V5 hnRNP E1 protein levels in LM2 cells treated with cycloheximide for 0 and 8 h. Hsp90 is used as a loading control. **k** Proliferation of LM2 cells stably expressing WT, K314R and K351R V5 hnRNP E1, as assessed by cell count. **l** Representative images of 2D invasion assay using LM2 cells stably expressing WT, K314R, and K351R V5 hnRNP E1.

ARIH1 modulation affected EMT induction and invasion in mammary epithelial cells. We next wanted to test the effect of ARIH1 modulation in breast cancer cells. Initially, we silenced ARIH1 by shRNA in the SUM159 cell line (Supplementary Fig. S2d). ARIH1 KD led to a decrease in the mesenchymal markers N-cadherin and Vimentin (Fig. 4a), an increase in hnRNP E1 protein levels (Supplementary Fig. S2e) and a reduction in cell invasion (Fig. 4b), with no change in cell proliferation (Supplementary Fig. S2f), as compared to scrambled control cells. Next, we assessed cancer stemness characteristics by mammosphere assay and PCR of CSC markers. Of note, silencing of ARIH1 led to a near complete loss of mammosphere forming capability and correlated with a reduction in CSC markers, including Oct4 and Sox2 (Fig. 4b–d). Overexpression of ARIH1 ORF in the SUM159 cell line led to a decrease in hnRNP E1 expression and increased mesenchymal markers (N-cadherin and Vimentin) (Supplementary Fig. S3a). Increased invasion in both 2D and 3D assays was also observed (Supplementary Fig. S3b, c), however, no change in mammosphere formation was detected (Supplementary Fig. S3d). To support our findings, we silenced ARIH1 in a second mesenchymal-like TNBC line, the lung metastases derivative of the MDA-MB-231 cell line, known as LM2-4175 (LM2; Supplementary Fig. S2g). Similar to our findings in SUM159 cells, ARIH1 KD in LM2-4175 cells reduced cell invasion (Fig. 4e), increased hnRNP E1 protein stability (Fig. 4f), and had no effect on cell proliferation (Supplementary Fig. S2h). We also observed decreased expression of the mesenchymal marker Vimentin with ARIH1 KD (Fig. 4g); in our hands, the LM2 line does not express N-cadherin or E-cadherin, therefore these markers are not included.

As hnRNP E1 is a known regulator of EMT and cancer progression, we tested the contribution of hnRNP E1 to the ARIH1 KD phenotype in our breast cancer model. To do this, hnRNP E1 was silenced using CRISPR/Cas9 technology in LM2 ARIH1 sh8 and SUM159 ARIH1 sh5 cells. In the LM2 ARIH1 sh8 cell line, hnRNP E1 silencing did not alter cell proliferation (Supplementary Fig. S4b) but did increase cell invasion, as assessed by 2D invasion assay (Fig. 4h, Supplementary Fig. S4e) and increased expression of the mesenchymal marker Vimentin and the stemness marker CD44 (Fig. 4g, Supplementary Fig. S4a), when compared to hnRNP E1 WT control. Of note, LM2 ARIH1 sh8 hnRNP E1 CRISPR cells were less invasive than LM2 scrambled control cells expressing ARIH1 (Supplementary Fig. S4f), indicating that hnRNP E1 partially contributes to the loss of invasion induced by ARIH1 KD in this cell line. In contrast, in the SUM159 cell line we observed reduced cell proliferation with hnRNP E1 KO with no effect on cell invasion or mammosphere formation (Supplementary Fig S4g-j).

Next, we expressed WT, K314R, and K351R hnRNP E1 in LM2 and SUM159 cells. Expression of V5 hnRNP E1 was markedly higher in both transient and stable cells when either lysine 314 or 351 was mutated (Fig. 4i, Supplementary Fig. S4k–m). Similar to our findings in NMUMG cells (Fig. 1e), V5 hnRNP E1 demonstrated increased stability when K314 or K351 was mutated to arginine as assessed by cycloheximide chase assay (Fig. 4j, Supplementary Fig. S4n). Moreover, we observed a marked increase in WT V5 hnRNP E1 protein levels upon silencing of ARIH1 by siRNA, and the effect of ARIH1 siRNA was attenuated in cells expressing K314R V5 hnRNP E1 (Supplementary Fig. S4o).

LM2 cells expressing K314R or K351R constructs had high levels of hnRNP E1 protein and demonstrated reduced expression of the mesenchymal marker Vimentin, and reduced invasion, when compared to empty vector control or WT V5 hnRNP E1 expressing cells (Fig. 4i, l). No change in cell proliferation was observed with either WT or mutant hnRNP E1 expression (Fig. 4k). Expression of K314R or K351R hnRNP E1 in the SUM159 line reduced mammosphere formation and invasion with no change in cell proliferation (Supplementary Fig. S4p–r). In this line, WT V5 hnRNP E1 was expressed at a higher level than in LM2 cells, and WT hnRNP E1 expression was capable of reducing cell invasion (Supplementary Fig. S4l, r). Overall, these data indicate that hnRNP E1 contributes to ARIH1’s function in EMT, stemness and cancer progression, however, the degree of contribution appears to be cell type dependent. These findings are consistent with our hypothesis that ARIH1 modulates breast cancer progression by modulating several targets, including hnRNP E1, in a coordinated manner.

**Fig. 5:**
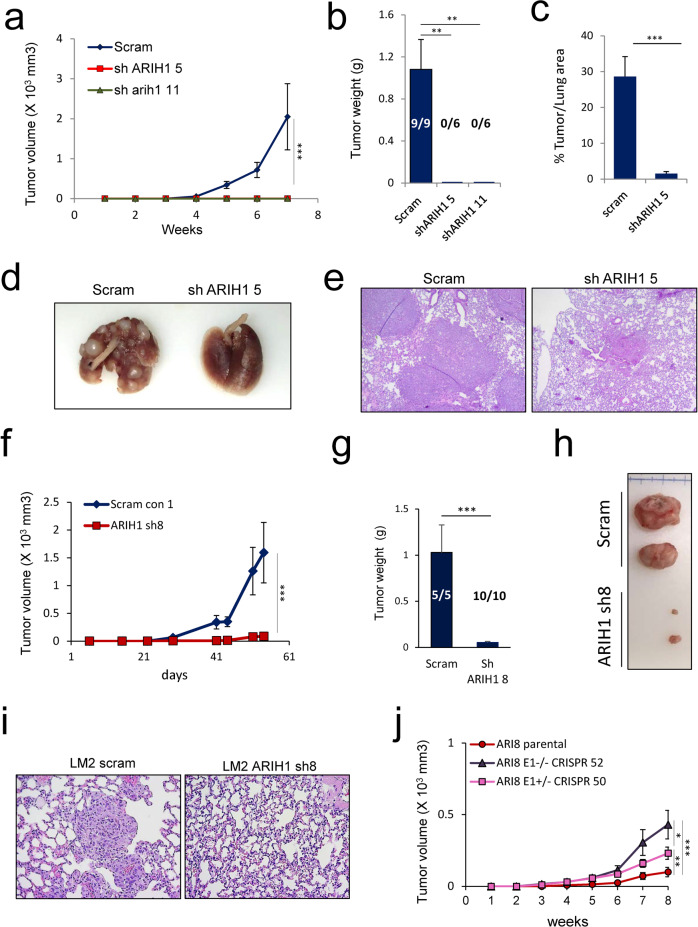
*ARIH1 modulates cancer progression* in vivo. **a** Tumor volumes in NOD-SCID mice mammary fat pad injected with SUM159 cells stably expressing scrambled control or ARIH1 shRNA (left panel, mean ± SEM, two-way ANOVA with Bonferroni post-hoc test; ***P < 0.001). **b** Quantitation of tumor weight and number of tumors that developed following xenograft of SUM159 cells stably expressing scrambled control or ARIH1 shRNA (*n* = 9 xenografts in scram control group, *n* = 6 xenografts per sh 5 and sh 11 group; mean + SEM, unpaired *t*-test compared to scram; **P < 0.01). **c** Quantitation of % tumor/lung area from H&E stained lung sections from NOD-SCID mice tail vein injected with SUM159 cells stably expressing scrambled control or ARIH1 shRNA (*n* = 4 in scram control group, *n* = 5 in ARIH1 KD group; mean + SEM, unpaired *t*-test compared to scram; ***P < 0.001). Representative images of (**d**) whole lungs and (**e**) H&E stained lung sections from tail vein injected mice. **f** Tumor volumes in NOD-SCID mice mammary fat pad injected with LM2 cells stably expressing scrambled control or ARIH1 shRNA (mean ± SEM, two-way ANOVA with Bonferroni post-hoc test; ***P < 0.001). **g** Quantitation of tumor weight and number of tumors that developed following xenograft of LM2 cells stably expressing scrambled control or ARIH1 shRNA (*n* = 5 xenografts in scram control group, *n* = 10 xenografts per sh 8 group; mean + SEM, unpaired *t*-test compared to scram; ***P < 0.001). Representative images of (**h**) tumors and (**i**) H&E staining of lung sections following xenograft of LM2 cells stably expressing scrambled control or ARIH1 shRNA. **j** Tumor volumes in NOD-SCID mice mammary fat pad injected with LM2 ARIH1 KD cells and LM2 ARIH1 KD hnRNP E1 CRISPR clone 50 and 52 (mean ± SEM, two-way ANOVA with Bonferroni post-hoc test; *P < 0.05, **P < 0.01, ***P < 0.001).

To test ARIH1 function in cancer progression in vivo, ARIH1-modulated SUM159 cells were injected into the mammary fat pad of NOD-SCID mice and tumor volumes were assessed over time. Xenograft injection of two ARIH1 shRNA clones into the mammary fat pad of NOD-SCID mice resulted in no tumors observed at 7 weeks, at which point mice injected with scrambled control cells had reached a maximum tumor burden of 2000 mm^3^ (Fig. 5a, b, Supplementary Fig. S3e). A subgroup of mice injected with ARIH1 KD cells were monitored for tumor burden past this time point, with no palpable tumors observed at 12 weeks. Consistent with this data, tail vein injection of ARIH1 KD SUM159 cells in NOD-SCID mice led to a significant reduction in lung colonization when compared to scrambled control injected mice (Fig. 5c–e). In contrast to ARIH1 silenced cells, xenograft injection of control and ARIH1 ORF SUM159 cells resulted in no significant change in tumor burden between groups (Supplementary Fig. S3f–h), consistent with our mammosphere assays performed in vitro (Fig. 4b, c, Supplementary Fig. S3d). Like our findings in SUM159 ARIH1 KD cells, xenograft injection of LM2 ARIH1 KD cells into the mammary fat pad of NOD-SCID mice resulted in significantly reduced tumor formation and the loss of lung metastases (Fig. 5f–i). The contribution of hnRNP E1 to the ARIH1 KD phenotype observed in LM2 mammary fat pad xenografts was tested using LM2 ARIH1 KD E1 CRISPR clones. We observed an increase in tumor volume and weight in xenografts from ARIH1 KD cells silenced for hnRNP E1, with hnRNP E1^−/−^ cells (clone 52) leading to increased tumor volume when compared to hnRNP E1^+/−^ cells (clone 50; Fig. 5j). This data is consistent with our invasion and immunoblot data showing increased invasion and expression of the mesenchymal marker Vimentin and stemness marker CD44 in hnRNP E1^−/−^ cells compared to hnRNP E1^+/−^ and parental hnRNP E1^+/+^ cells (Fig. 4g, h, Supplementary Fig. S4a).

**Fig. 6:**
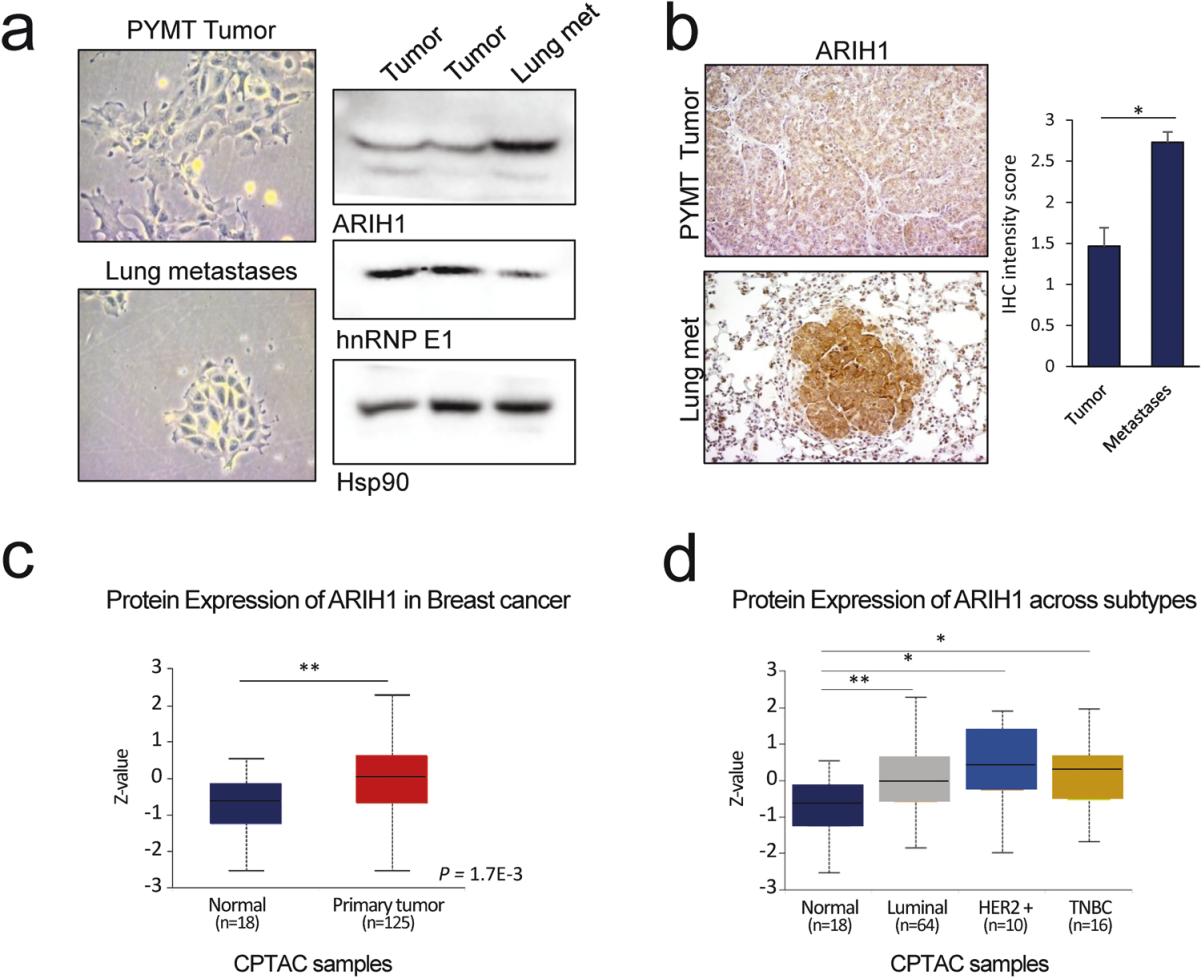
*ARIH1 expression correlates with cancer progression*. **a** Cell morphology and immunoblot analyses of hnRNP E1 and ARIH1 protein levels in cell cultured from primary mammary tumors and lung metastases isolated from the MMTV-PyMT mouse model. Hsp90 protein was used as a loading control. **b** Immunohistochemical analyses of ARIH1 expression in PyMT mammary tumors and matched lung metastases. Quantitation of ARIH1 IHC intensity score (1 = low, 2 = moderate, 3 = high staining intensity) in mammary tumors and matched lung metastases (*n* = 5; mean + SEM, paired t-test, *P < 0.05). Protein expression of ARIH1 in (**c**) normal and tumor breast tissue and (**d**) across breast cancer subtypes.

To further characterize the role of ARIH1 in cancer progression in vivo, we utilized the MMTV-PyVT (PyMT) mouse model of breast cancer. An increase in ARIH1 expression was observed in cells isolated from lung metastases, compared to mammary tumors from PyMT mice. Furthermore, decreased hnRNP E1 protein levels were detected in these metastatic cells (Fig. 6a). To further interrogate ARIH1 expression in this model, we performed immunohistochemistry on five matched mammary tumor and lung specimens. ARIH1 staining was observed in both the cytoplasm and nucleus of tumor cells and a significant increase in stain intensity was observed in lung metastases when compared to primary tumor (Fig. 6b). Finally, to investigate ARIH1’s role in human breast cancer, we interrogated data from the Clinical Proteomic Tumor Analysis Consortium (CPTAC) using the UALCAN platform [19]. A significant increase in ARIH1 protein expression was observed in tumor versus normal breast tissue, with no association with cancer subtype (Fig. 6c, d). Moreover, high ARIH1 expression was associated with reduced survival in breast cancer patients (Supplementary Fig. S5a, b). In support of a link between ARIH1 expression and EMT induction, analysis of human breast cancer proteome data revealed an inverse correlation between ARIH1 protein levels and levels of the epithelial markers E-cadherin and Occludin (Supplementary Fig S5c, d).

ARIH1’s function in tumorigenesis does not appear to be limited to breast cancer. A similar increase in ARIH1 protein expression was observed in lung adenocarcinoma samples, with ARIH1 expression significantly correlating with tumor grade (Supplementary Fig. S5e, f). Moreover, the effect of ARIH1 silencing on cell invasion was observed in colorectal cancer cells. Increased ARIH1 and reduced hnRNP E1 protein levels were detected in the more aggressive HCT116 colorectal cancer cell line, compared to SW480, SW620, and HCT8 cells (Supplementary Fig. S6a). Knockdown of ARIH1 in the HCT116 line did not alter cell proliferation but did reduce cell invasion in a 3D invasion assay (Supplementary Fig. S6b–d). Moreover, ARIH1 protein expression in colorectal cancer patients positively correlated with Vimentin protein expression and negatively correlated with E-cadherin (Supplementary Fig. S6e), strengthening the link between ARIH1 and EMT induction in cancer. Overall, these data highlight a novel role for ARIH1 in tumorigenesis.

### Novel interactors may contribute to ARIH1’s role in EMT and cancer progression

**Fig. 7:**
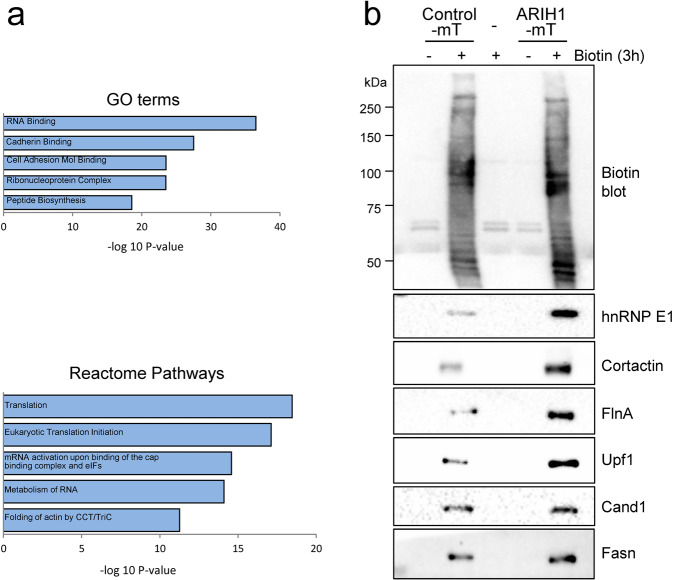
*Novel interactors of ARIH1*. Pathway analyses of proteins purified from biotin-treated, ARIH1-miniTurboID transfected Hek293 cells. **a** Enriched biological processes from gene ontology and reactome pathways, identified using the Molecular Signature Database (MSigDB). **b** Immunoblot of biotin, hnRNP E1, UPF1, Cortactin, Filamin A (FlnA), Cand1 and Fasn in Hek293 cells either mock transfected or transfected with empty vector miniTurboID or ARIH1-miniTurboID and treated with or without biotin for 3 h.

To further interrogate the mechanisms through which ARIH1 modulates EMT induction and cancer progression, we utilized the miniTurboID system to purify ARIH1-interacting protein and analyzed control (no biotin) and biotin treated samples by mass spectrometry. This data was filtered by removing common hits between control and biotin-treated samples that included biotinylated carboxylases such as ACACA, PC, MCCC, and PCC. We identified 74 genes that were enriched in biotin-treated ARIH1 pulldowns, including hnRNP E1/PCBP1, in addition to UBAP2L and UPF1, which have been previously characterized as high confidence ARIH1 interactors (Supplementary Table S2) [13]. Pathway analyses revealed an enrichment of proteins involved in translation initiation and proteins associated with cadherin binding and cell adhesion molecule binding (Fig. 7a). Such factors included Filamin A, Cortactin, Talin 1 and TJP1 (ZO1) that act as scaffolds to regulate cytoskeletal dynamics [20–23], a process that is modulated during the transition to a mesenchymal phenotype. Multiple EMT-associated proteins were also identified, including UPF1 [24], CYLD [25, 26], and AHNAK [27, 28]. Moreover, in addition to hnRNP E1, the RNA binding proteins hnRNPF, hnRNPH1, and hnRNPM were identified [29–31].

Next, we independently validated several hits in Hek293 cells transiently expressing either control or ARIH1 miniTurboID. This analysis validated UPF1, Filamin A and Cortactin as ARIH1 interactors (Fig. 7b), whereas, similar pulldown levels of Cand1 and Fasn were observed between control and ARIH1 miniTurboID samples, suggesting non-specific pulldown (Fig. 7b). Overall, these data suggest that ARIH1 regulates EMT induction, stemness and cancer progression through the coordinated control of multiple downstream targets. As ARIH1’s function in a cancer setting has not been adequately explored, the identification of novel interactors of this ligase may lead to significant advancements in our understanding of this protein’s role in tumorigenesis.

## Discussion

Despite the well-defined role of hnRNP E1 as a tumor suppressor that functions in EMT and cancer progression, little is known about the regulation of this RNA binding protein. Here, we describe the post-translational regulation of hnRNP E1 mediated by the ubiquitin E3 ligase ARIH1. Our data is consistent with previous work that demonstrates a reduction in hnRNP E1 protein levels in cancer compared to normal tissue [32–34]. In studies where both mRNA and protein levels of hnRNP E1 are compared, mRNA levels that are expressed across samples do not correlate with protein levels suggesting a post-transcriptional mechanism of regulation [34, 35]. Consistent with this hypothesis, a recent study in prostate cancer cells demonstrated a reduction in hnRNP E1 protein upon TGFβ treatment, which was reported to be due to hnRNP E1 ubiquitination and subsequent degradation [36]. Furthermore, the proteasome inhibitor MG132 increased hnRNP E1 protein in thyroid cancer cell lines with low levels of hnRNP E1 protein despite abundant mRNA expression [35]. Overall these data indicate regulation of protein stability as one mechanism to control hnRNP E1 expression.

In terms of post-translational modifications, one mechanism of hnRNP E1 control is via phosphorylation at sites including S43, T60, and T127 [6, 37]. This modification induces nuclear localization of hnRNP E1 leading to a switch from translational repression to transcriptional and splicing regulation. In addition to phosphorylation, several lysine residues on hnRNP E1 are ubiquitinated with K23, K314, and K351 observed across multiple studies curated in the PhosphoSitePlus database [18]. In our model, we observed an increase in protein stability and reduced ubiquitination of hnRNP E1 when lysine 314 was mutated to arginine (Fig. 1e, f), indicating that modification of this residue by ubiquitin plays an important role in hnRNP E1 protein stability. It will be interesting to test whether such ubiquitination modulates localization or function of hnRNP E1 and whether phosphorylation of hnRNP E1 alters ubiquitination of this protein.

We observe an increase in ARIH1 expression upon TGFβ treatment (Fig. 2a, b), and we hypothesize that this upregulation is in part due to increased transcript abundance. Analysis of the ARIH1 3’UTR using TargetScan 7.2 [38] revealed conserved miRNA binding sites for the miR-200bc/429 cluster, as well as miR-219-5p and miR-124-3p. These miRNAs play an important role in EMT suppression and are repressed during the transition to the mesenchymal phenotype [39–41], thus relieving suppression of their targets which may include ARIH1. Our data suggest an additional post-translational mechanism of ARIH1 control, based on the observation that Flag-ARIH1 ORF is increased following TGFβ treatment in over-expressing cells (Supplementary Fig. S3a). Indeed, post-transcriptional control of ARIH1 has been described previously; NF2 has been shown to alter ARIH1 protein levels with no significant change in mRNA level [42]. Moreover, YAP inhibition is reported to reduce ARIH1 protein expression [42]. In a separate study, ARIH1 protein levels increased in U20S cells following DNA damage, and data indicated that this increase was due to a reduction in ARIH1 protein degradation [17].

ARIH1 functions in a number of processes including mitophagy, myonuclear anchoring and DNA damage-induced translation arrest. In the context of tumorigenesis, ARIH1 upregulation in cancer and an association with the proliferation marker ki67 have been described previously [15, 43]. However, ARIH1’s function in EMT induction and cancer progression has not been defined. Here, we demonstrate that ARIH1 is upregulated during TGFβ-induced EMT and this E3 ubiquitin ligase plays a novel role in the mesenchymal transition and cancer progression. Our data suggest that hnRNP E1 partially contributes to ARIH1’s function in these processes, and the degree of contribution appears to be cell type dependent. We observe reduced stability of endogenous and WT V5 hnRNP E1 in LM2 cells compared to SUM159 cells (Fig. 4f, j, Supplementary Figs. S2e, S4m & n). Moreover, our data indicates that K314R increases protein stability of hnRNP E1 more than K351R, and we observe that expression of K314R hnRNP E1 is higher in LM2 cells when compared to K351R (Fig. 4i), whereas, only a slight difference between these mutants is observed in SUM159 cells (Supplementary Fig. S4l & m). Thus, we propose that the different outcomes observed upon hnRNP E1 modulation in LM2 and SUM159 cells are due to differences in hnRNP E1 protein stability between these lines.

We hypothesize that ARIH1’s role in EMT and cancer progression is a result of modulating several targets in a coordinated manner. Consistent with this hypothesis, in ARIH1 miniTurboID experiments we detect several interacting proteins with a role in EMT modulation and cancer progression, including TJP1 (ZO1), CYLD, and UPF1 (Supplementary Table S2). Overall, further research is warranted to interrogate ARIH1 expression regulation under both physiological and pathological conditions and to delineate the mechanisms through which this ligase functions during EMT and cancer progression.

## Materials and methods

### Cell lines and treatments

NMuMG, SUM cells, LM2-4175, HEK293, HCT116, HCT8, SW620, SW480, and Lenti-X 293 T cell lines were cultured in DMEM high glucose supplemented with 10% Serum and 1% antibiotic/antimycotic solution (penicillin G, streptomycin, amphotericin B) at 37 °C, 5% CO_2._ HMLE Epi cells were isolated and cultured as described previously [44]. NMuMG, HEK293, and colorectal cells were obtained from the American Type Culture Collection (ATCC). Lenti-X 293 T cells were obtained from Clontech. SUM cell lines were kindly provided by Dr. Ethier (Medical University of South Carolina). LM2-4175 cells were kindly provided by Dr. Massague (Memorial Sloan-Kettering Cancer Center). Cycloheximide, biotin, and PR619 were purchased from Sigma-Aldrich, MLN4924 from Abcam, and MG132 from Cayman Chemicals.

### Animal studies

All animal procedures were approved by the Animal Care and Use Committees of the Medical University of South Carolina. Nonobese diabetic, severe combined immunodeficient (NOD/SCID) mice (NOD.CB17-Prkdc^SCID^/J) were supplied by Envigo. No randomization was used to select animals for each group and studies were not blinded. Mammary fat pad injection of 1 × 10^6^ SUM159 cells or 5 × 10^5^ LM2 cells or tail vein injection of 5 × 10^5^ cells was performed using 6–9 week old NOD/SCID females. For mammary fat pad experiments, tumor volumes (mm^3^) were measured weekly using digital calipers and tumors were weighted at experimental endpoints. For lung colonization experiments, formalin fixed, paraffin embedded lung sections were cut at 5 μm and stained with hematoxylin and eosin (H&E) for histopathological evaluation by the Biorepository and Tissue Analysis Resource at MUSC. Micrographs of stained sections were taken using a Leica DMIL LED microscope with Amscope camera and acquisition software. Lung and metastases area were determined using ImageJ software in order to calculate tumor/lung ratios (%). FVB/N-Tg(MMTV-PyVT)634Mul/J males purchased from the Jackson laboratory were crossed with FVB/N females to produce experimental females hemizygous for MMTV-PyMT. PyMT mammary tumors and lungs were collagenase treated to dissociate, followed by centrifugation and resuspension in a 1:4 mixture of cold HF (Hanks’ Balanced Salt Solution supplemented with 2% FBS) and Ammonium Chloride Solution. Partially-dissociated tissue samples were trypsinized in 0.05% Trypsin-EDTA, followed by 2 mL of pre-warmed Dispase (1 U/mL) and 20 μL of DNase I Solution (1 mg/mL). Cell suspensions were filtered through 40 μm cell strainers, centrifuged at 350 x g for 5 min and seeded using mammary epithelial media (DMEM/F-12 supplemented with 10 mM HEPES, 10 μg/ml Insulin, 1X Glutamax and B27, 10 ng/ml EGF and FGF, 4 μg/ml Heparin and penicillin/streptomycin) plus 5% FBS. A media change in serum free mammary epithelial media was performed 24 h post seeding.

### miniTurbo BioID assay

Human ARIH1 ORF was cloned into V5-miniTurbo-NES_pCDNA3 (Plasmid #107170 Addgene), a gift from Alice Ting [45]. Hek293 cells were transiently transfected with either empty vector miniTurboID or ARIH1-miniTurboID and treated with 100 μM biotin (Sigma) 3 h prior to harvesting in RIPA lysis buffer. Lysates were incubated with streptavidin agarose beads (Invitrogen) overnight at 4 °C with rotation. Beads were washed three times in RIPA buffer and proteins were eluted by heat denaturation at 95 °C for 5 min in Laemmli buffer. Mass spectrometry analyses were performed by the Taplin Biological Mass Spectrometry Facility at Harvard Medical School.

### Lentiviral transductions

Stable knockdown cells were generated by lentiviral transduction of pLKO.1 puro vectors (Sigma) containing human ARIH1 shRNA (TRCN0000007500), mouse ARIH1 shRNA or a scrambled control sequence (CCTAAGGTTAAGTCGCCCTCG). Stable overexpression pools of V5-tagged hnRNP E1 (HsCD00435748; DNASU) or Flag-tagged ARIH1 (EX-U1404-Lv101; GeneCopoeia) were generated through lentiviral transduction and stable selection of cells.

### WT and mutant V5 hnRNP E1 expression in SUM159 and LM2 cells

Nucleofection of empty vector, WT and mutant V5 hnRNP E1 in SUM159 and LM2 cells was performed using an Amaxa nucleofector and protocol X-013 (LM2) or V-001 (SUM159). Stable pools were selected and characterized.

### CRISPR/Cas9

hnRNP E1 KO in SUM159 and LM24175 cells was performed using the AltR CRISPR/Cas9 system (IDT). Nucleofection of two guides (GATGCCGGTGTGACTGAAAG and CTCCATGACCAACAGTACCG) and AltR Cas9 enzyme into cells was performed using an Amaxa nucleofector and protocol X-013 (LM2) or V-001 (SUM159). Edited clones were identified by PCR (primers detailed in Supplementary Table S1) and further characterized by immunoblot and Sanger sequencing using CRISP-ID software [46] (Supplementary Table S3).

### Site directed mutagenesis

Site directed mutagenesis was performed using the QuickChange Lightning site directed mutagenesis kit (Agilent Technologies) with primers listed in Supplementary data (Supplementary Table S1).

### siRNA transfections

Cells were transfected using 25 nM of a scrambled control siRNA or ARIH1 siRNA (CGAGAUAUUUCCCAAGAUU; ON-TARGETplus siRNA, Thermo Fisher) and lipofectamine 3000 as per manufacturer’s instructions. Media changes were performed 4-24 h post transfection.

### 2D and 3D invasion assays

2D invasion was performed using BD BioCoat™ Matrigel™ Invasion Chambers (BD Biosciences) or QCM ECMatrix colorimetric Cell invasion assays (Millipore Sigma), as per manufacturer’s instructions. 3D Invasion was assessed using a modified Trevigen spheroid invasion assay as described previously [47]. The amount of Cultrex matrix used was modified based on the invasiveness of the cell line (50 μl Cultrex was used for HCT116 and LM2 cells, 37.5 μl for SUM159 cells and 25 μl for NMuMG and HMLE Epi cells).

### Yeast 2 hybrid assay

Human hnRNP E1 was cloned into the yeast two-hybrid DNA-binding domain vector pGBT9 (Clontech). The plasmid was transformed into yeast strain PJ69-4A [48]. The PJ69-4A strain containing pGBT9-hnRNP E1 was mated with yeast strain Y187 containing a normalized library of HeLa cell cDNAs cloned into a GAL4 AD vector (Clontech, Takara). The resulting library in the diploid strain was screened for activation of the ADE2 reporter gene on yeast minimal medium lacking leucine, tryptophan, and adenine. Positive clones were confirmed for interaction by further tests in the PJ69-4A strain and plasmid DNA was sequenced to identify interacting genes.

### V5 hnRNP E1 immunoprecipitation

NMuMG cells stably expressing V5-tagged hnRNP E1 were transfected with 1 μg HA-Ubiquitin plasmid (Plasmid #18712 Addgene). Cells were harvested 48 h post transfection and were treated with MG132 4 h prior to harvest. Cells were lyzed in IP lysis buffer (50 mM Tris-HCl pH 7.4, 150 mM NaCl, 1 mM EDTA, 1 mM EGTA, 0.5% NP-40, 1% Triton X-100, protease/phosphatase inhibitor cocktail, 10 μM PR619) and kept on ice for 30 min prior to centrifugation at 14,000 g for 15 min. Cells stably expressing WT or lysine mutant V5-tagged hnRNP E1 were treated with MG132 4 h prior to harvesting under denaturing conditions using IP lysis buffer containing 1% SDS, heat denaturing at 95 °C for 5 min, followed by dilution of SDS to 0.1% using IP lysis buffer prior to centrifugation and IP. Protein estimation of cleared cell lysates was performed by Bradford assay. 1–2 mg of lysates was incubated with anti-V5 antibody overnight at 4 °C with rotation. Lysate:antibody solution was incubated with protein A Sepharose 4B beads (ThermoFisher) for 2 h at 4 °C with rotation. Beads were washed 4 times in IP lysis buffer and protein eluted by heat denaturation at 95 °C for 5 min in Laemmli buffer.

### Immunoblotting

Immunoblot analysis was performed as described previously [47]. Briefly, cells were lysed in RIPA buffer and denatured at 95 °C for 5 min in Laemmli buffer. Samples were resolved on SDS–PAGE gels and transferred to PVDF membrane. Blots were probed with antibodies specific to ARIH1 (VPA00397; BioRad), hnRNP E1 (M01; Abnova), E-cadherin (3195; Cell Signaling), N-cadherin (BD Transductions), Vimentin (5741; Cell Signaling), Biotin (5597; Cell Signaling), Flag (14793; Cell Signaling), K48 Ubiquitin (8081; Cell Signaling), K63 Ubiquitin (5621; Cell Signaling), Nedd8 (2745; Cell Signaling), Hsp90 (sc13119: Santa Cruz Biotechnology), CD44 (GTX102111; GeneTex), V5 (R96025; ThermoFisher), UPF1 (VMA00627; BioRad), Filamin A (VMA00322; BioRad), Cortactin (VMA00430; BioRad), CAND1 (VMA00610; BioRad), and FASN (VMA00266; BioRad). Chemiluminescence was detected by CCD camera (BioRad ChemiDoc system).

### MTT assay

MTT assay was performed as described previously [47], with cell lines seeded at 10^3^ cells per well of a 96 well plate.

### Semi-quantitative PCR

RNA isolation and semi-quantitative PCR was performed as described previously [44]. Primer sequences are described in Supplementary data (Supplementary Table S1).

### Immunohistochemistry

ARIH1 staining of mouse mammary tumor and lung tissue were performed by the Biorepository and Tissue Analysis core at MUSC as described previously [47], using ARIH1 antibody (PA5-51885; Invitrogen). Micrographs were taken using a Leica DMIL LED microscope with Amscope camera and acquisition software.

### Immunofluorescence

Immunofluorescence analyses were performed as described previously [47]. The following primary antibodies were used: E-cadherin (3195: Cell Signaling), Vimentin (5741; Cell Signaling) using either Alexa Fluor 488- or 568-conjugated secondary antibodies (Thermo Fisher). For Phalloidin immunofluorescence, rhodamine phalloidin (Thermo Fisher) was used. Images were taken using an Olympus FV10i LIV laser scanning confocal microscope.

### Mammosphere assay

Mammosphere assays were performed as described previously [47].

### Statistical analyses

All data are presented as the mean ± SD, unless otherwise stated and representative experiments were repeated at least twice. Statistical analyses using SPSS software were performed by two-tailed Student’s t-test of pair-wise comparisons or ANOVA with Bonferroni post-hoc test for group comparisons. Spearman or Pearson correlation coefficients were determined for CPTAC colorectal and breast proteome data using cBioPortal [49] and Log Rank (Mantel cox) test for Kaplan–Meier analyses. *P*-values <0.05 were considered statistically significant.

## Supplementary information

Supplemental material
