# Supplementary material for: The ubiquitin E3 ligase ARIH1 regulates hnRNP E1 protein stability, EMT and breast cancer progression
Source: Oncogene. 2022 Jan 31;41(12):1679–90. doi: 10.1038/s41388-022-02199-9 (PMC8933277; doi:10.1038/s41388-022-02199-9)
Supplement: Supplementary file 1 — Supplemental material [file 41388_2022_2199_MOESM1_ESM.pdf]

Supplemental Figure legends

Figure S1. (a) Expression of hnRNP E1 transcript levels in NMuMG cells treated with TGFβ. βActin was used as a loading control. **(b)** Polysome profiling of NMuMG cells either untreated or treated with TGFβ for 7 days. hnRNP E1 is actively translated under both conditions, whereas, the positive control inhibin βA shifts to heavier polysome fractions following TGFβ treatment. **(c)** Immunoblot of hnRNP E1 and Hsp90 (loading control) following treatment of NMuMG cells with cycloheximide for 12h and co-treatment with either the ER-Golgi trafficking inhibitor Brefeldin A, the lysosome inhibitor chloroquine or the proteasome inhibitor MG132. **(d)** Interaction between hnRNP E1 and ARIH1 in yeast 2 hybrid assay. Growth observed on Leu-/Trp-/Ade- plates indicating interaction between hnRNP E1 and ARIH1. **(e)** Immunoblot of hnRNP E1, ARIH1 and Hsp90 (loading control) following ARIH1 siRNA in NMuMG cells. **(f)** hnRNP E1 protein levels following treatment with the neddylation inhibitor MLN4924. **(g)** Immunoprecipitation of exogenous V5 tagged hnRNP E1 followed by K63 ubiquitin immunoblot. **(h)** Protein stability, assessed by cycloheximide chase assay, of WT and K314R or K351R mutant V5-tagged hnRNP E1 with or without MG132 treatment.

Figure S2. (a) Transcript levels of the epithelial marker, E-cadherin, in NMuMG cells treated with TGFβ, as determined by semi-quantitative PCR. βActin was used as a loading control. **(b)** Rhodamine phalloidin staining of actin in NMuMG control and ARIH1 KD cells following 2 d of TGFβ treatment. **(c)** 3D invasion assay of NMuMG control and ARIH1 KD cells; representative images of invasion in Matrigel, invasive areas are delineated. **(d)** Immunoblot of ARIH1 expression levels in SUM159 control and ARIH1 ORF cells and SUM159 scrambled control and ARIH1 KD clones (sh5 and sh11). Hsp90 was used as a loading control. **(e)** Protein levels of hnRNP E1 in cycloheximide-treated SUM159 scrambled control and ARIH1 KD cells. Hsp90 is used as a loading control. **(f)** Proliferation of SUM159 WT, scrambled control and ARIH1 KD cells, as assessed by MTT assay. **(g)** Immunoblot of ARIH1 expression levels in LM2 scrambled control

(scram) and ARIH1 sh8 KD cells. Hsp90 was used as a loading control. **(h)** Proliferation rates of LM2 scrambled control and ARIH1 KD cells.

Figure S3. **(a)** Protein levels of hnRNP E1 and the mesenchymal markers N-Cadherin and Vimentin in TGFβ-treated SUM159 control and ARIH1 ORF cells. **(b)** 2D invasion assay of SUM159 control and ARIH1 ORF cells; representative images of invasive cells (left panel) and quantitation (Mean ± SD, unpaired t-test compared to control; * = $P < 0.05$). **(c)** 3D invasion assay of SUM159 control and ARIH1 ORF cells; representative images of invasion in Matrigel. **(d)** Mammosphere assay of SUM159 control and ARIH1 ORF cells; representative images of spheres and quantitation of sphere number (Mean ± SEM, paired t-test compared to control was not significant). **(e)** Image of excised tumors from mice injected with SUM159 cells stably expressing scrambled control. **(f)** Image of excised tumors from mice injected with SUM159 control and ARIH1 ORF cells. **(g)** Tumor volumes in NOD-SCID mice mammary fat pad injected with SUM159 control and ARIH1 ORF cells (mean ± SEM). **(h)** Quantitation of tumor weight following xenograft of SUM159 control and ARIH1 ORF cells (n = 9 control xenografts, n = 7 ORF xenografts; mean + SEM, unpaired t-test; n.s = not significant).

Figure S4. **(a)** Protein levels of Vimentin and CD44 in LM2 scrambled control, ARIH1 Sh8 KD cells and ARIH1 sh8 KD with hnRNP E1 silencing (CRISPR 50 and 52). **(b)** Proliferation rates of LM2 scrambled control cells, ARIH1 sh8 KD cells and ARIH1 sh8 KD cells with hnRNP E1 silencing. **(c)** Representative image of excised tumors and **(d)** tumor weights from mice injected with LM2 ARIH1 sh8 E1 CRISPR 50 and 52. **(e)** Quantitation of 2D invasion, following crystal violet stain extraction, using LM2 ARIH1 KD cells and ARIH1 KD with hnRNP E1 silencing (mean +SD). **(f)** Representative images of 3D invasion assay with LM2 scrambled control cells and ARIH1 KD cells with hnRNP E1 silencing. **(g)** Protein levels of hnRNP E1 in SUM159 ARIH1 KD cells with hnRNP E1 CRISPR. **(h)** Proliferation rates of parental SUM159 ARIH1 KD cells and ARIH1 KD cells with hnRNP E1 CRISPR. **(i)** Representative

images of 3D invasion assay with SUM159 scrambled control cells, ARIH1 KD cells and ARIH1 KD cells with hnRNP E1 silencing. **(j)** Mammosphere formation in SUM159 scrambled control cells, ARIH1 KD cells and ARIH1 KD cells with hnRNP E1 silencing. V5 hnRNP E1 protein levels in **(k)** SUM159 ARIH1 ORF cells 3 d post transient nucleofection, and **(l)** stably selected SUM159 cells. V5 hnRNP E1 protein levels in stably selected SUM159 cells treated with cycloheximide for **(m)** 0, 4 and 8 h and for **(n)** 0 and 17 h. Hsp90 is used as a loading control. **(o)** V5 hnRNP E1 protein levels in stably selected SUM159 cells transiently tranfected with control or ARIH1 siRNA for 72 h. **(p)** Proliferation of SUM159 cells stably expressing WT, K314R and K351R V5 hnRNP E1, as assessed by cell count. **(q)** Representative images and quantitation of mammosphere number (Mean ± SEM, paired t-test compared to control; ** = $P < 0.01$, *** = $P < 0.001$). **(r)** Representative images of 2D invasion assay using SUM159 cells stably expressing WT, K314R and K351R V5 hnRNP E1.

Figure S5. (a) Kaplan-Meier analysis of survival in TCGA breast cancer patient data. (n = 1194, TCGA dataset; P=0.05, log-rank test). **(b)** Kaplan-Meier analysis of relapse-free survival in breast cancer patients. (n = 3951, KM-plotter; P<0.001, log-rank test). Correlation between ARIH1 protein levels and the epithelial markers **(c)** E-cadherin and **(d)** Occuldin in breast cancer patient samples analyzed using cBioPortal. **(e)** Protein expression of ARIH1 in normal lung and lung adenocarcinoma tissue. **(f)** Protein expression of ARIH1 across lung cancer grade.

Figure S6. (a) Protein levels of hnRNP E1 and ARIH1 across a panel of colorectal cancer cell lines. Hsp90 was used as a loading control (top panel). hnRNP E1 transcript levels are not altered across these lines, as assessed by semi-quantitative PCR. GAPDH was used as a loading control (bottom panel). **(b)** Representative images of HCT116 scrambled control and ARIH1 KD cell morphology. **(c)** Proliferation of HCT116 scrambled control and ARIH1 KD cells, as assessed by cell count across 4 days. **(d)** 3D invasion assay of HCT116 WT, scrambled control and ARIH1 KD cells; representative images of invasion in Cultrex. **(e)** Correlation between ARIH1 protein

levels and the mesenchymal marker Vimentin (left panel) and the epithelial marker E-Cadherin (right panel) in colorectal cancer patient samples analyzed using cBioPortal.

Table S1. Sequences and expected product size of primers used for PCR.

Table S2. List of proteins identified from ARIH1-miniTurboID experiment.

Table S3. Sanger sequencing results for hnRNP E1 CRISPR clones.

Supplemental Figure 1

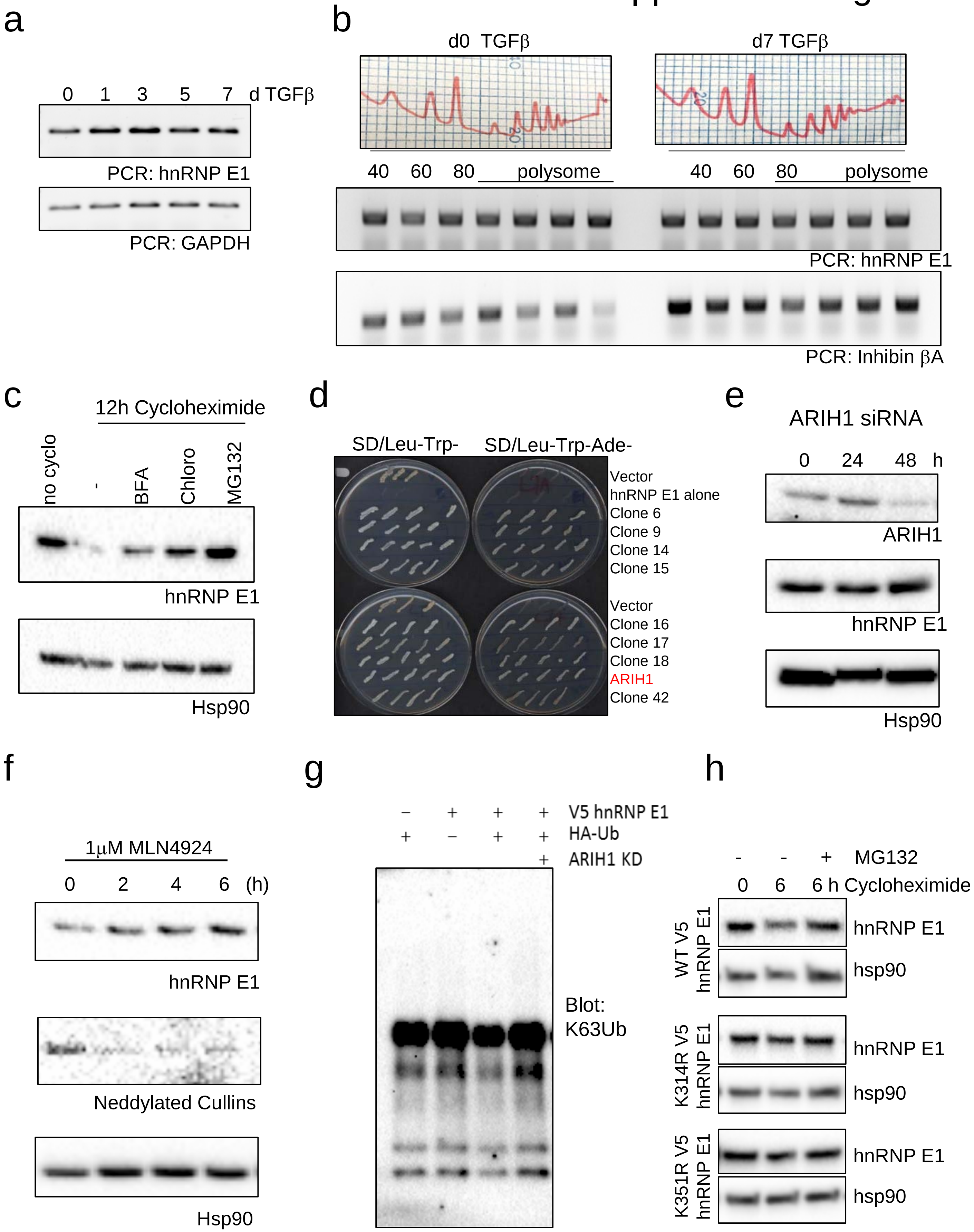

Supplemental Figure 2

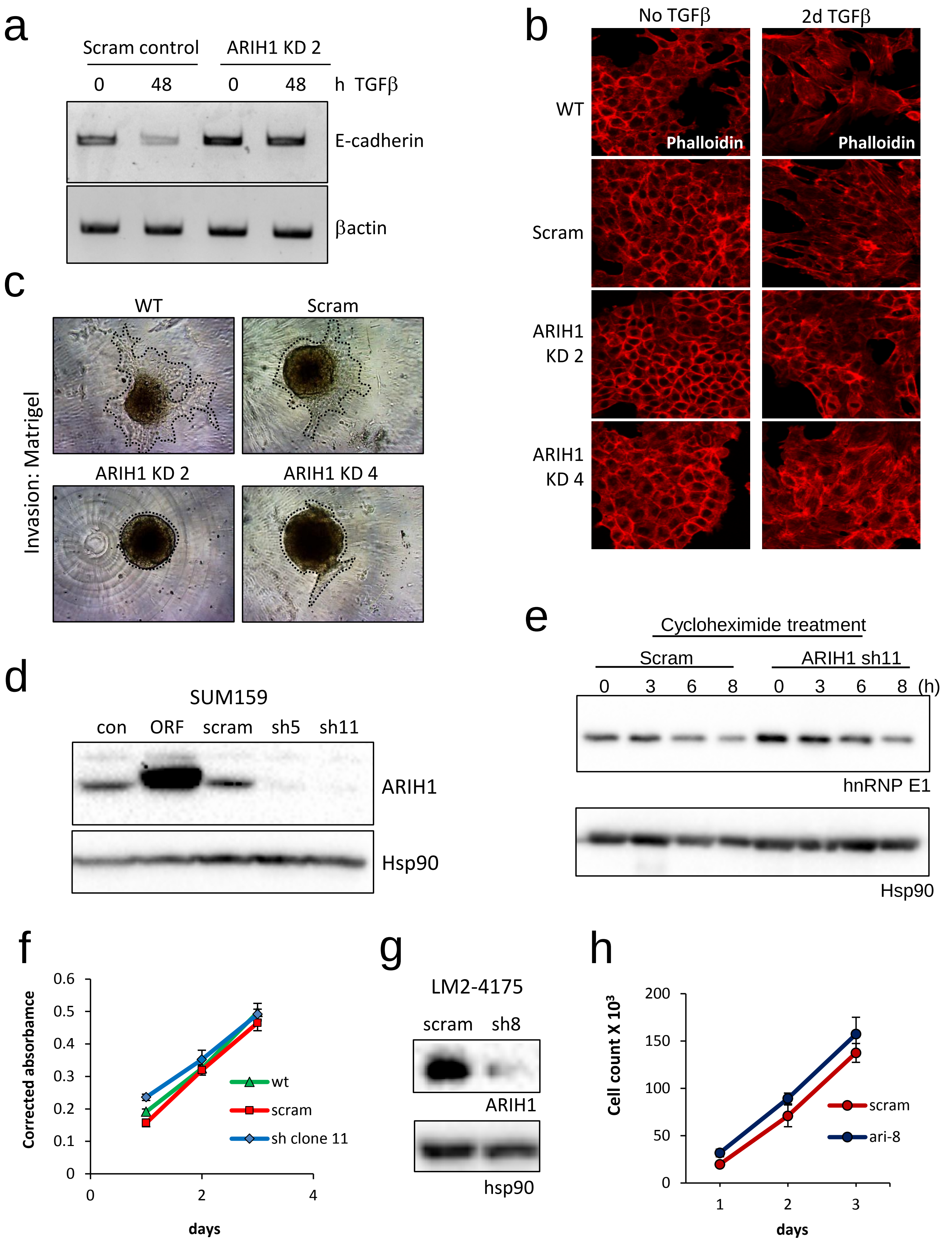

Supplemental Figure 3

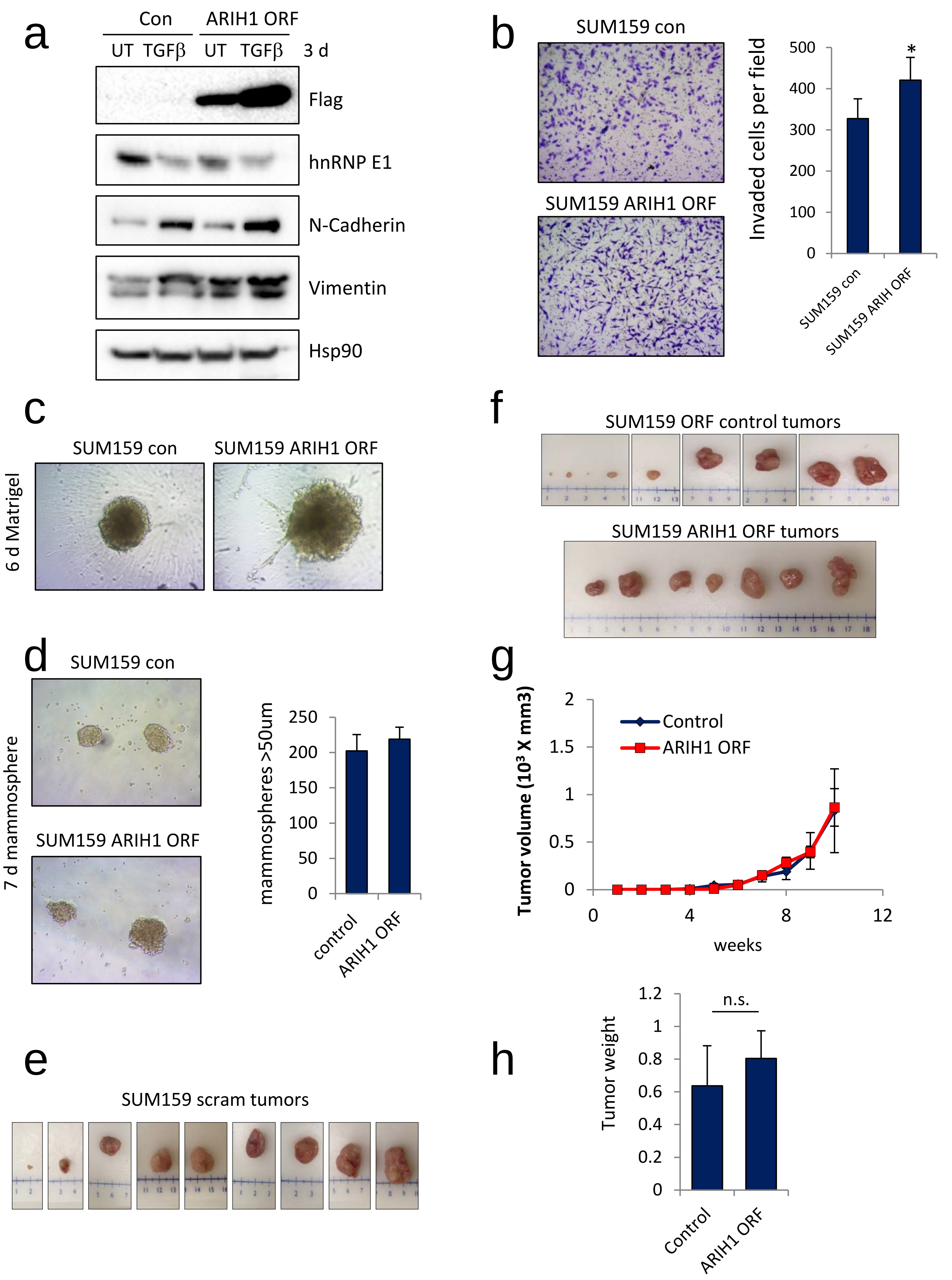

Supplemental Figure 4

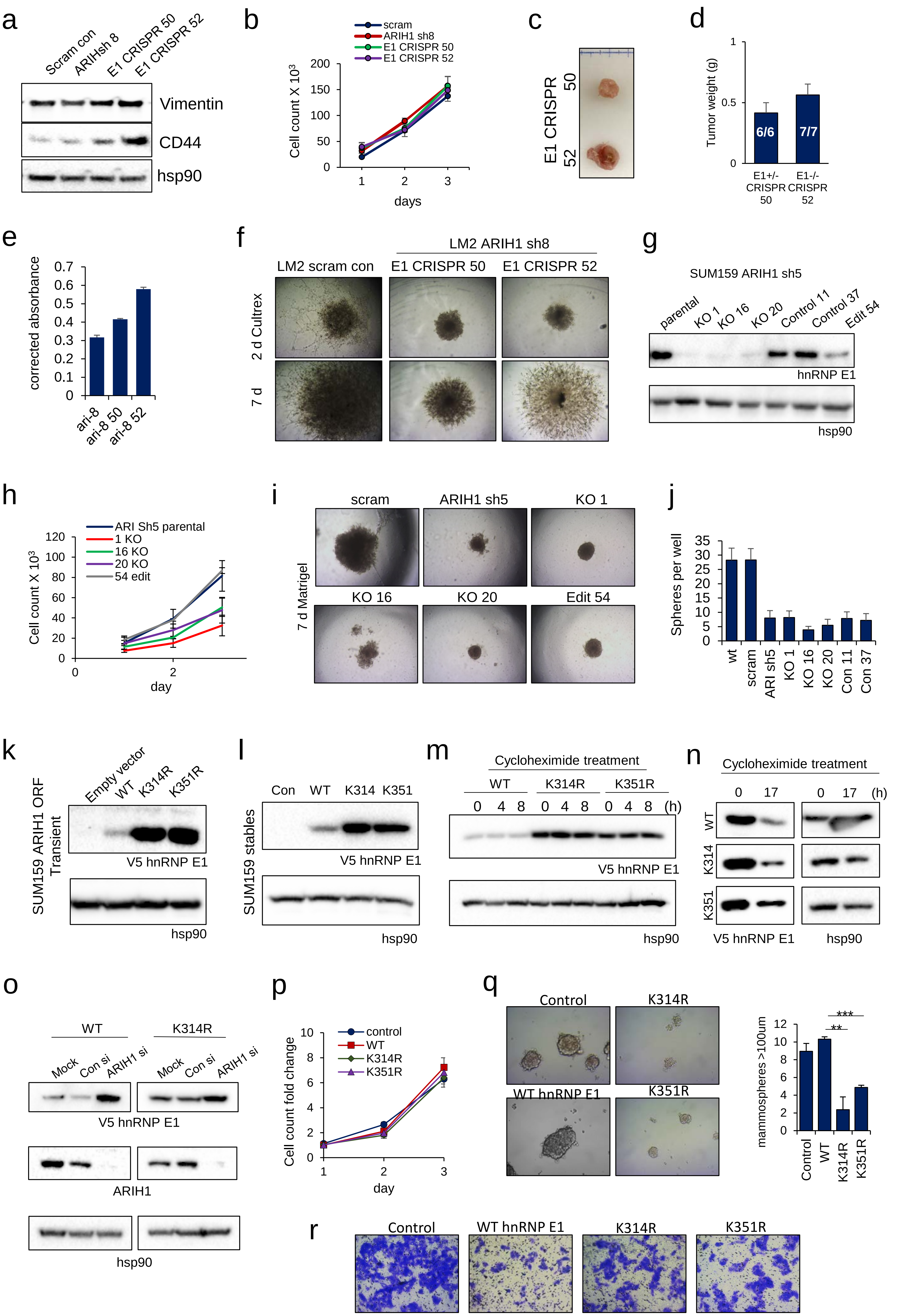

Supplemental Figure 5

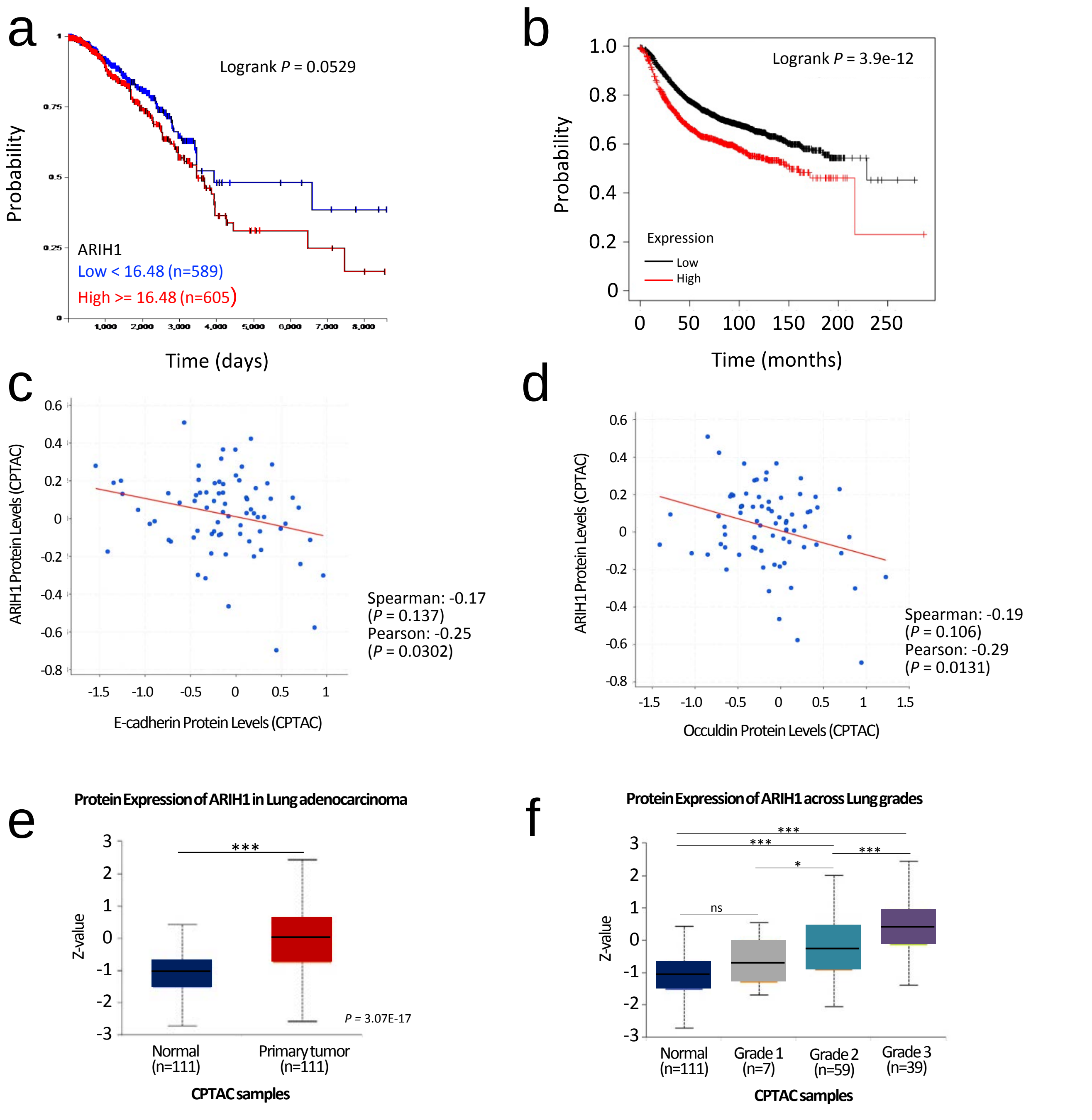

Supplemental Figure 6

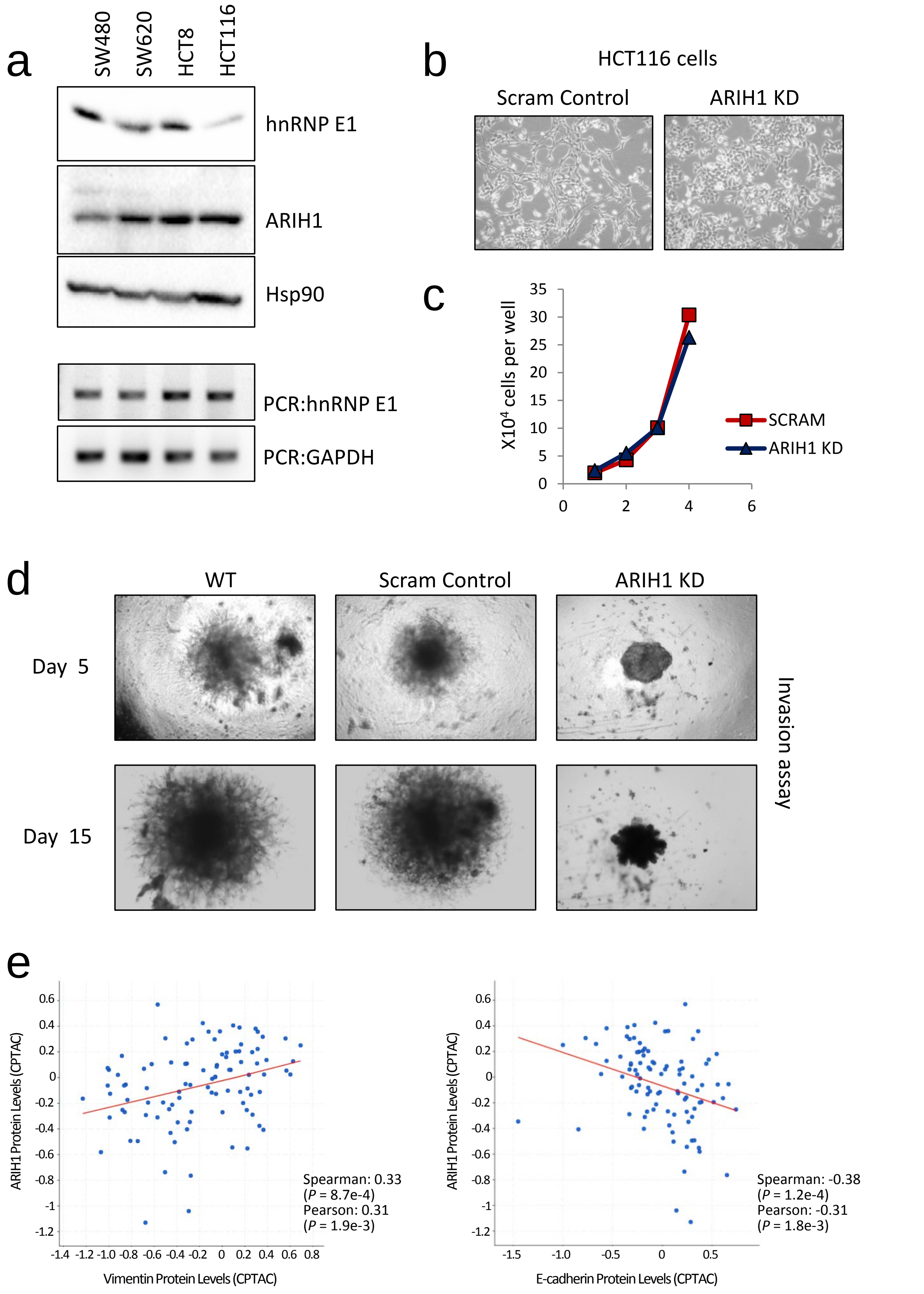

Table S1. Primers used for PCR

**Target**	**Sequence**	**Product size**
Mouse hnRNP E1	Forward: AAGGCGCCAACATCAATGAG; Reverse: GGGTTACGAGCGAACCTACG	204 bp
Mouse ARIH1	Forward:GAGGTCATCCAGAATCCAGCA ; Reverse:TGCGCAGGACACGAAATAGT	338 bp
Mouse E-Cadherin	Forward: GAAGGCTTGAGCACAACAGC; Reverse: AGATGGGGGCTTCATTCACG	400 bp
Mouse Inhibin βA	Forward: TCCCCCACCCCAGGATCCGA; Reverse: ACCGGCTGGGTGACATCGGG	173 bp
Mouse GAPDH	Forward: GGTTGTCTCCTGCGACTTCA; Reverse: TAGGGCCTCTCTTGCTCAGT	220 bp
Human hnRNP E1	Forward: CCACGTAACGAGCCCAACT; Reverse: CCCTCCGAGATGTTGATCCG	200 bp
Human ARIH1	Forward: AGAGAATGCCACAGAGGTGC; Reverse: CCCTTCGTCGACTCTCACAG	119 bp
Human Aldh1a1	Forward: ATCAAAGAAGCTGCCGGGAA; Reverse: GCATTGTCCAAGTCGGCATC	101 bp
Human Klf4	Forward: ATGCTCACCCCACCTTCTTC; Reverse: GGTGGTCCGACCTGGAAAAT	340 bp
Human Oct4	Forward: CTCACCCTGGGGGTTCTATT; Reverse: CTGGTTCGCTTTCTCTTTCG	203 bp
Human Sox2	Forward: AACCAGCGCATGGACAGTTA; Reverse: GACTTGACCACCGAACCCAT	278 bp
Human Nanog	Forward: AAAGGCAAACAACCCACTTC; Reverse: CATTGCTATTCTTCGGCCAG	274 bp
Human GAPDH	Forward: TGATGACATCAAGAAGGTGGTGAAG; Reverse: TCCTTGGAGGCCATGTGGGCCAT	240 bp
β−Actin	Forward: TCATGAAGTGTGACGTTGACATCCGT; Reverse: CCTAGAAGCATTTGCGGTGCACGATG	285 bp
miniturboID ARIH1	Forward: GGTGGTAAGCTTATGGACTCGGACGAGGGCTACA;<br>Reverse: TGCTTAGCGGCCGCGTCCTCAATGTACTCCCACA	1.6 kb
hnRNP E1 CRISPR primer set A	Forward: ACTTGACCACGTAACGAGCC; Reverse: TCCAGCATGACCAGGCAAAT	554 bp
hnRNP E1 CRISPR primer set B	Forward: TCGGCTTCTTATGCACGGAA; Reverse: CTTCCTCCAGCTTGTCGATGA	197 bp
K351 site directed mut. primers	Forward: CCCCATGCCCCTCTCAGAGGAAAGCCTGGC;<br>Reverse: GCCAGGCTTTCCTCTGAGAGGGGCATGGGG	n/a
K314 site directed mut. primers	Forward: CTGGGTTGGCAATTCTGATCTGGGCCCCG;<br>Reverse: CGGGGCCCAGATCAGAATTGCCAACCCAG	n/a

Table S2. Proteins identified from ARIH1-miniTurboID experiment

reference	Gene Symbol	Annotation	Unique hits: Hek_ARIH1 mTbioID_Con	Total hits: Hek_ARIH1 mTbioID_Con	Unique hits: Hek_ARIH1 mTbioID_Biotin	Total hits: Hek_ARIH1 mTbioID_Biotin
sp|P21333|FLNA_HUMAN	FLNA	Filamin-A OS=Homo sapiens GN=FLNA PE=1 SV=4	0	0	51	56
sp|P49327|FAS_HUMAN	FASN	Fatty acid synthase OS=Homo sapiens GN=FASN PE=1 SV=3	0	0	21	21
sp|P50990|TCPQ_HUMAN	CCT8	T-complex protein 1 subunit theta OS=Homo sapiens GN=CCT8 PE=1 SV=4	0	0	16	16
sp|Q04637|IF4G1_HUMAN	EIF4G1	Eukaryotic translation initiation factor 4 gamma 1 OS=Homo sapiens GN=EIF4G1 PE=1 SV=4	0	0	9	10
sp|P11388|TOP2A_HUMAN	TOP2A	DNA topoisomerase 2-alpha OS=Homo sapiens GN=TOP2A PE=1 SV=3	0	0	7	7
sp|Q99613|EIF3C_HUMAN	EIF3C	Eukaryotic translation initiation factor 3 subunit C OS=Homo sapiens GN=EIF3C PE=1 SV=1	0	0	7	7
sp|Q6WCQ1|MPRIP_HUMAN	MPRIP	Myosin phosphatase Rho-interacting protein OS=Homo sapiens GN=MPRIP PE=1 SV=3	0	0	7	7
sp|P46060|RAGP1_HUMAN	RANGAP1	Ran GTPase-activating protein 1 OS=Homo sapiens GN=RANGAP1 PE=1 SV=1	0	0	7	7
sp|Q09666|AHNK_HUMAN	AHNAK	Neuroblast differentiation-associated protein AHNAK OS=Homo sapiens GN=AHNAK PE=1 SV=2	0	0	7	7
sp|P27816|MAP4_HUMAN	MAP4	Microtubule-associated protein 4 OS=Homo sapiens GN=MAP4 PE=1 SV=3	0	0	7	7
sp|P37802|TAGL2_HUMAN	TAGLN2	Transgelin-2 OS=Homo sapiens GN=TAGLN2 PE=1 SV=3	0	0	6	7
sp|P52907|CAZA1_HUMAN	CAPZA1	F-actin-capping protein subunit alpha-1 OS=Homo sapiens GN=CAPZA1 PE=1 SV=3	0	0	5	6
sp|Q9UM54|MYO6_HUMAN	MYO6	Unconventional myosin-VI OS=Homo sapiens GN=MYO6 PE=1 SV=4	0	0	5	5
sp|Q86VP6|CAND1_HUMAN	CAND1	Cullin-associated NEDD8-dissociated protein 1 OS=Homo sapiens GN=CAND1 PE=1 SV=2	0	0	5	5
sp|Q9Y4X5|ARI1_HUMAN	ARIH1	E3 ubiquitin-protein ligase ARIH1 OS=Homo sapiens GN=ARIH1 PE=1 SV=2	0	0	4	4
sp|Q14152|EIF3A_HUMAN	EIF3A	Eukaryotic translation initiation factor 3 subunit A OS=Homo sapiens GN=EIF3A PE=1 SV=1	0	0	4	4
sp|Q6Y7W6|GGYF2_HUMAN	GIGYF2	GRB10-interacting GYF protein 2 OS=Homo sapiens GN=GIGYF2 PE=1 SV=1	0	0	4	4
sp|P40227|TCPZ_HUMAN	CCT6A	T-complex protein 1 subunit zeta OS=Homo sapiens GN=CCT6A PE=1 SV=3	0	0	4	4
sp|Q9Y5K6|CD2AP_HUMAN	CD2AP	CD2-associated protein OS=Homo sapiens GN=CD2AP PE=1 SV=1	0	0	4	4
sp|O75367|H2AY_HUMAN	H2AFY	Core histone macro-H2A.1 OS=Homo sapiens GN=H2AFY PE=1 SV=4	0	0	4	4
sp|P49368|TCPG_HUMAN	CCT3	T-complex protein 1 subunit gamma OS=Homo sapiens GN=CCT3 PE=1 SV=4	0	0	4	4
sp|Q00341|VIGLN_HUMAN	HDLBP	Vigilin OS=Homo sapiens GN=HDLBP PE=1 SV=2	0	0	3	3
sp|Q9Y490|TLN1_HUMAN	TLN1	Talin-1 OS=Homo sapiens GN=TLN1 PE=1 SV=3	0	0	3	3
sp|Q9P0K7|RAI14_HUMAN	RAI14	Ankycorbin OS=Homo sapiens GN=RAI14 PE=1 SV=2	0	0	3	3
sp|O75821|EIF3G_HUMAN	EIF3G	Eukaryotic translation initiation factor 3 subunit G OS=Homo sapiens GN=EIF3G PE=1 SV=2	0	0	3	3
sp|P49411|EFTU_HUMAN	TUFM	Elongation factor Tu, mitochondrial OS=Homo sapiens GN=TUFM PE=1 SV=2	0	0	3	3
sp|Q92900|RENT1_HUMAN	UPF1	Regulator of nonsense transcripts 1 OS=Homo sapiens GN=UPF1 PE=1 SV=2	0	0	3	3
sp|Q58FF7|H90B3_HUMAN	HSP90AB3P	Putative heat shock protein HSP 90-beta-3 OS=Homo sapiens GN=HSP90AB3P PE=5 SV=1	0	0	3	3
sp|Q16512|PKN1_HUMAN	PKN1	Serine/threonine-protein kinase N1 OS=Homo sapiens GN=PKN1 PE=1 SV=2	0	0	3	3
sp|P49959|MRE11_HUMAN	MRE11	Double-strand break repair protein MRE11 OS=Homo sapiens GN=MRE11 PE=1 SV=3	0	0	3	3
sp|Q15365|PCBP1_HUMAN	PCBP1	Poly(rC)-binding protein 1 OS=Homo sapiens GN=PCBP1 PE=1 SV=2	0	0	2	3
sp|P52597|HNRPF_HUMAN	HNRNPF	Heterogeneous nuclear ribonucleoprotein F OS=Homo sapiens GN=HNRNPF PE=1 SV=3	0	0	2	3
sp|Q14157|UBP2L_HUMAN	UBAP2L	Ubiquitin-associated protein 2-like OS=Homo sapiens GN=UBAP2L PE=1 SV=2	0	0	2	2
sp|P50914|RL14_HUMAN	RPL14	60S ribosomal protein L14 OS=Homo sapiens GN=RPL14 PE=1 SV=4	0	0	2	2
sp|O00303|EIF3F_HUMAN	EIF3F	Eukaryotic translation initiation factor 3 subunit F OS=Homo sapiens GN=EIF3F PE=1 SV=1	0	0	2	2
sp|P07814|SYEP_HUMAN	EPRS	Bifunctional glutamate/proline--tRNA ligase OS=Homo sapiens GN=EPRS PE=1 SV=5	0	0	2	2
sp|Q13765|NACA_HUMAN	NACA	Nascent polypeptide-associated complex subunit alpha OS=Homo sapiens GN=NACA PE=1 SV=1	0	0	2	2
sp|Q14247|SRC8_HUMAN	CTTN	Src substrate cortactin OS=Homo sapiens GN=CTTN PE=1 SV=2	0	0	2	2
sp|Q15393|SF3B3_HUMAN	SF3B3	Splicing factor 3B subunit 3 OS=Homo sapiens GN=SF3B3 PE=1 SV=4	0	0	2	2
sp|P60866|RS20_HUMAN	RPS20	40S ribosomal protein S20 OS=Homo sapiens GN=RPS20 PE=1 SV=1	0	0	2	2
sp|P31943|HNRH1_HUMAN	HNRNPH1	Heterogeneous nuclear ribonucleoprotein H OS=Homo sapiens GN=HNRNPH1 PE=1 SV=4	0	0	2	2
sp|P17661|DESM_HUMAN	DES	Desmin OS=Homo sapiens GN=DES PE=1 SV=3	0	0	2	2
sp|P78527|PRKDC_HUMAN	PRKDC	DNA-dependent protein kinase catalytic subunit OS=Homo sapiens GN=PRKDC PE=1 SV=3	0	0	2	2
sp|P14868|SYDC_HUMAN	DARS	Aspartate--tRNA ligase, cytoplasmic OS=Homo sapiens GN=DARS PE=1 SV=2	0	0	2	2
sp|P57737|CORO7_HUMAN	CORO7	Coronin-7 OS=Homo sapiens GN=CORO7 PE=1 SV=2	0	0	2	2
sp|P63173|RL38_HUMAN	RPL38	60S ribosomal protein L38 OS=Homo sapiens GN=RPL38 PE=1 SV=2	0	0	2	2
sp|P13010|XRCC5_HUMAN	XRCC5	X-ray repair cross-complementing protein 5 OS=Homo sapiens GN=XRCC5 PE=1 SV=3	0	0	2	2
sp|P63244|RACK1_HUMAN	RACK1	Receptor of activated protein C kinase 1 OS=Homo sapiens GN=RACK1 PE=1 SV=3	0	0	2	2
sp|Q15019|SEPT2_HUMAN	SEPT2	Septin-2 OS=Homo sapiens GN=SEPT2 PE=1 SV=1	0	0	2	2
sp|Q16555|DPYL2_HUMAN	DPYSL2	Dihydropyrimidinase-related protein 2 OS=Homo sapiens GN=DPYSL2 PE=1 SV=1	0	0	2	2
sp|P50991|TCPD_HUMAN	CCT4	T-complex protein 1 subunit delta OS=Homo sapiens GN=CCT4 PE=1 SV=4	0	0	2	2
sp|O95347|SMC2_HUMAN	SMC2	Structural maintenance of chromosomes protein 2 OS=Homo sapiens GN=SMC2 PE=1 SV=2	0	0	2	2
sp|P78344|IF4G2_HUMAN	EIF4G2	Eukaryotic translation initiation factor 4 gamma 2 OS=Homo sapiens GN=EIF4G2 PE=1 SV=1	0	0	2	2
sp|Q9NQC7|CYLD_HUMAN	CYLD	Ubiquitin carboxyl-terminal hydrolase CYLD OS=Homo sapiens GN=CYLD PE=1 SV=1	0	0	2	2
sp|A6NNT2|CP096_HUMAN	C16orf96	Uncharacterized protein C16orf96 OS=Homo sapiens GN=C16orf96 PE=4 SV=3	0	0	2	2
sp|Q07157|ZO1_HUMAN	TJP1	Tight junction protein ZO-1 OS=Homo sapiens GN=TJP1 PE=1 SV=3	0	0	2	2
sp|P33176|KINH_HUMAN	KIF5B	Kinesin-1 heavy chain OS=Homo sapiens GN=KIF5B PE=1 SV=1	0	0	2	2
sp|P62851|RS25_HUMAN	RPS25	40S ribosomal protein S25 OS=Homo sapiens GN=RPS25 PE=1 SV=1	0	0	2	2
sp|P11021|GRP78_HUMAN	HSPA5	78 kDa glucose-regulated protein OS=Homo sapiens GN=HSPA5 PE=1 SV=2	1	1	5	5
sp|P17987|TCPA_HUMAN	TCP1	T-complex protein 1 subunit alpha OS=Homo sapiens GN=TCP1 PE=1 SV=1	1	1	5	5
sp|P62701|RS4X_HUMAN	RPS4X	40S ribosomal protein S4, X isoform OS=Homo sapiens GN=RPS4X PE=1 SV=2	1	1	4	5
sp|P54652|HSP72_HUMAN	HSPA2	Heat shock-related 70 kDa protein 2 OS=Homo sapiens GN=HSPA2 PE=1 SV=1	1	1	4	4
sp|Q14240|IF4A2_HUMAN	EIF4A2	Eukaryotic initiation factor 4A-II OS=Homo sapiens GN=EIF4A2 PE=1 SV=2	1	1	4	4
sp|O94832|MYO1D_HUMAN	MYO1D	Unconventional myosin-Id OS=Homo sapiens GN=MYO1D PE=1 SV=2	1	1	4	4
sp|P13639|EF2_HUMAN	EEF2	Elongation factor 2 OS=Homo sapiens GN=EEF2 PE=1 SV=4	1	1	3	4
sp|P16403|H12_HUMAN	HIST1H1C	Histone H1.2 OS=Homo sapiens GN=HIST1H1C PE=1 SV=2	1	1	3	3
sp|P06576|ATPB_HUMAN	ATP5B	ATP synthase subunit beta, mitochondrial OS=Homo sapiens GN=ATP5B PE=1 SV=3	1	1	3	3
sp|P26373|RL13_HUMAN	RPL13	60S ribosomal protein L13 OS=Homo sapiens GN=RPL13 PE=1 SV=4	2	2	6	6
sp|P17844|DDX5_HUMAN	DDX5	Probable ATP-dependent RNA helicase DDX5 OS=Homo sapiens GN=DDX5 PE=1 SV=1	2	2	4	5
sp|P34931|HS71L_HUMAN	HSPA1L	Heat shock 70 kDa protein 1-like OS=Homo sapiens GN=HSPA1L PE=1 SV=2	3	3	9	10
sp|P0DMV9|HS71B_HUMAN	HSPA1B	Heat shock 70 kDa protein 1B OS=Homo sapiens GN=HSPA1B PE=1 SV=1	3	3	8	8
sp|P19338|NUCL_HUMAN	NCL	Nucleolin OS=Homo sapiens GN=NCL PE=1 SV=3	3	3	7	7
sp|P09874|PARP1_HUMAN	PARP1	Poly [ADP-ribose] polymerase 1 OS=Homo sapiens GN=PARP1 PE=1 SV=4	4	4	13	13
sp|P52272|HNRPM_HUMAN	HNRNPM	Heterogeneous nuclear ribonucleoprotein M OS=Homo sapiens GN=HNRNPM PE=1 SV=3	4	4	10	10

Table S3. Sanger sequencing of hnRNP E1 CRISPR clones

Legend: Guide RNA, Ref = ARIH1 KD parental sequencing results.

LM2-4175 ARIH1 sh8 hnRNP E1 CRISPR clone 50 product (550 bp)

```
Ref   TCGCGGATCTCTTTGATCTTACACCCGCCTTTCCCAATCAGGGAGCCGCACTGGGTGGCCGGCACCACCAGCCTCAGGGTGACCGGGGGCCTGCTGGCCG
Seq 1 TCGCGGATCTCTTTGATCTTACACCCGCCTTTCCCAATCAGGGAGCCGCACTGGGTGGCCGGCACCACCAGCCTCAGGGTGACCGGGGGCCTGCTGGCCG
Seq 2 TCGCGGATCTCTTTGATCTTACACCCGCCTTTCCCAATCAGGGAGCCGCACTGGGTGGCCGGCACCACCAGCCTCAGGGTGACCGGGGGCCTGCTGGCCG

Ref   CGGTACTGTTGGTCATGGAGCTGTTGATATCTTCCTCCAGCTTGTCGATGATCATAGCGAAAGCCTTAAAGATGGCATTGGTGGGGCCGGTCAGAGTGAT
Seq 1 CGGTACTGTTGGTCATGGAGCTGTTGATATCTTCCTCCAGCTTGTCGATGATCATAGCGAAAGCCTTAAAGATGGCATTGGTGGGGCCGGTCAGAGTGAT
Seq 2 CGGTACTGTTGGTCATGGAGCTGTTGATATCTTCCTCCAGCTTGTCGATGATCATAGCGAAAGCCTTAAAGATGGCATTGGTGGGGCCGGTCAGAGTGAT

Ref   GATTCTCTCCGGACAATTCCCCTCCGAGATGTTGATCCGCGCGCCACTCTCCTCGCGGATCCTCTTAACCGACTCCCCTTTCTTCCCAATGATGCTTCCT
Seq 1 GATTCTCTCCGGACAATTCCCCTCCGAGATGTTGATCCGCGCGCCACTCTCCTCGCGGATCCTCTTAACCGACTCCCCTTTCTTCCCAATGATGCTTCCT
Seq 2 GATTCTCTCCGGACAATTCCCCTCCGAGATGTTGATCCGCGCGCCACTCTCCTCGCGGATCCTCTTAACCGACTCCCCTTTCTTCCCAATGATGCTTCCT

Ref   ACTTCCTTTCCGTGCATAAGAAGCCGAATGGTGAGAGTCACATTTAGTCCAC-TTTCAGTCACACCGGCATCCATGGCGAGCGGCGGGCGGCGTTCGGGG
Seq 1 ACTTCCTTTCCGTGCATAAGAAGCCGAATGGTGAGAGTCACATTTAGTCCAC-TT------ACACCGGCATCCATGGCGAGCGGCGGGCGGCGTTCGGGG
Seq 2 ACTTCCTTTCCGTGCATAAGAAGCCGAATGGTGAGAGTCACATTTAGTCCACTTTTCAGTCACACCGGCATCCATGGCGAGCGGCGGGCGGCGTTCGGGG
```

LM2-4175 ARIH1 sh8 hnRNP E1 CRISPR clone 52 product A (550 bp)

```
Ref   TCGCGGATCTCTTTGATCTTACACCCGCCTTTCCCAATCAGGGAGCCGCACTGGGTGGCCGGCACCACCAGCCTCAGGGTGACCGGGGGCCTGCTGGCCG
Seq 1 TCGCGGATCTCTTTGATCTTACACCCGCCTTTCCCAATCAGGGAGCCGCACTGGGTGGCCGGCACCACCAGCCTCAGGGTGACCGGGGGCCTGCTGGCCG
Seq 2 TCGCGGATCTCTTTGATCTTACACCCGCCTTTCCCAATCAGGGAGCCGCACTGGGTGGCCGGCACCACCAGCCTCAGGGTGACCGGGGGCCTGCTGGCCG
Seq 3 TCGCGGATCTCTTTGATCTTACACCCGCCTTTCCCAATCAGGGAGCCGCACTGGGTGGCCGGCACCACCAGCCTCAGGGTGACCGGGGGCCTGCTGGTGG

Ref   CGGTACTGTTGGTCATGGAGCTGTTGATATCTTCC------TC---CAGCTTGTCGATGATC-----------ATAGCGAAAGCCTTAAAGATGG---CA
Seq 1 CGGTGAAGGTGGTGATAGAAC---------CTTCCGA----T--------TTGTCCATCATC-----------TTTGCGAATGACTTAAAGAAAG---CC
Seq 2 CGGTGGCGGTGGTCAT--------TGA-ATCCTGCGAATTGTCCCTCATCTTGGCGAA--------AGACCTAA---CGAAAGCCTTG--AAGGGGACCG
Seq 3 CCGTGCTTCTGGGTATG-------TG------TGCG----GTCA-TCATCTTGTCGCAGCTCGCCGAGAT-TAATAGCAAAACACTAGAAAATGGAA---

Ref   TTGGTGGGGC-CGGTCA----GAGTGATG---ATTCTCTCC--GGACAATTCCCCTCCG--AGATGTTGATCCGCGCGCCACTCTCCTCGCGGATCCTCT
Seq 1 TTGAAGGGGA-CGGTTG----GAGGGGCG---GTTCTAACC--GAAAATTTCCCC--CGAAAAATGCCCATCCGAAAGGTACTCCCCTCCCCGATCCTCT
Seq 2 TTGGAGGGG--CGGT---TCTAAGGGGTG--ATTTC---CCCTGGAAAATTCCCCTCCGAAAGCTT---------------ATCCCCTCCCCCATCCTCT
Seq 3 -TGGGGGGGATCAGTCACTTTA-----TGCAATCTCTCGCCC----AAATCCTGTTCC--ACGCTTCGGATTCCCCGGCAACTCTCCTCGCCAATTCTCC

Ref   TAACCGACTCCCCTTTCTTCCCAATGATGCTTCC--TACTTCCTTTCCGTGCAT--------AAGAAGCCGAATG---GTGAGA-------G----T---
Seq 1 TAACGGACCCCCTTATCTTCCCCCCCTTGCTTCC--TACTGACGTTCCCTACTT--------CCTAACCCGAATG---AAGAGA-CCAACGG----TTAA
Seq 2 TACCGGACCCCCTTAACCACCCCCCTTTGCTTCCCA--ATTCTTTTCCTTGCTTC--------TTAACCCGAATG---AAGAGACCCATTG-----TTAA
Seq 3 TTCCGGA-----TT--------CCCGATGCTTCCAATGATGC---------CTTCGACTATAATTACCCGGATTGTAAAAGTGA---AATGGGGGATTAA

Ref   -----CACATTTAGTCCACTTTCAGTCACACCGGCATCCATGGCGAGCGGCGGG
Seq 1 AA-CACTCTTTTACTCCACTT-------------CATCCCCGGCGACCAGCGGG
Seq 2 AAGCCCTTTTTTA-TCCACTTCCT---CCA-----C-----GGCGTCCAGCGGG
Seq 3 AAC---------A-TCGACA-CCTGTGACATTGGTA-----GGCGT-----GGG
```

LM2-4175 ARIH1 sh8 hnRNP E1 CRISPR clone 52 product B (~300 bp)

```
Ref  TCGCGGATCTCTTTGATCTTACACCCGCCTTTCCCAATCAGGGAGCCGCACTGGGTGGCCGGCACCACCAGCCTCAGGGTGACCGGGGGCCTGCTGGCCG
Seq1 TCGCGGATCTCTTTGATCTTACACCCGCCTTTCCCAATCAGGGAGCCGCACTGGGTGGCCGGCACCACCAGCCTCAGGGTGACCGGGGGCCTGCTGGCCG

Ref  CGGTACTGTTGGTCATGGAGCTGTTGATATCTTCCTCCAGCTTGTCGATGATCATAGCGAAAGCCTTAAAGATGGCATTGGTGGGGCCGGTCAGAGTGAT
Seq1 CGGT------------------------------------------------------------------------------------------------

Ref  GATTCTCTCCGGACAATTCCCCTCCGAGATGTTGATCCGCGCGCCACTCTCCTCGCGGATCCTCTTAACCGACTCCCCTTTCTTCCCAATGATGCTTCCT
Seq1 ----------------------------------------------------------------------------------------------------

Ref  ACTTCCTTTCCGTGCATAAGAAGCCGAATGGTGAGAGTCACATTTAGTCCACTTTCAGTCACACCGGCATCCATGGCGAGC
Seq1 -------------------------------------------------------CAGTCACACCGGCATCCATGGCGAGC
```

SUM159 ARIH1 sh5 hnRNP E1 CRISPR clone 1 product A (550 bp)

```
Ref  TCGCGGATCTCTTTGATCTTACACCCGCCTTTCCCAATCAGGGAGCCGCACTGGGTGGCCGGCACCACCAGCCTCAGGGTGACCGGGGGCCTGCTGGCCG
Seq1 TCGCGGATCTCTTTGATCTTACACCCGCCTTTCCCAATCAGGGAGCCGCACTGGGTGGCCGGCACCACCAGCCTCAGGGTGACCGGGGGCCTGCTGGCCG
Seq2 TCGCGGATCTCTTTGATCTTACACCCGCCTTTCCCAATCAGGGAGCCGCACTGGGTGGCCGGCACCACCAGCCTCAGGGTGACCGGGGGCCTGCTGGCCG

Ref  CGGTACTGTTGGTCATGGAGCTGTTGATATCTTCCTCCAGCTTGTCGATGATCATAG-CGAAAGCCTTAAAGATGGCATTGGTGGGGCCGGTCAGAGTGA
Seq1 CGGTACTGTTGGTCATGGAGCTGTTGATATCTTCCTCCAGCTTGTCGATGATCATAG-CGAAAGCCTTAAAGATGGCATTGGTGGGGCCGGTCAGAGTGA
Seq2 CGGCCTTGCTGGCCAGCGTCCGGCGGAAATGGTGGGCGGCGTTGTGGGTACTCATAGCCGTAA-CCTGAAAGATGACATTGGTGGGGCCGGTCAGAGTGA

Ref  TGATTCTCTCCGGACAATTCCCCTCCGAGATGTTGATCCGCGCGCCACTCTCCTCGCGGATCCTCTTAACCGACTCCCCTTTCTTCCCAATGATGCTTCC
Seq1 TGATTCTCTCCGGACAATTCCCCTCCGAGATGTTGATCCGCGCGCCACTCTCCTCGCGGATCCTCTTAACCGACTCCCCTTTCTTCCCAATGATGCTTCC
Seq2 TGATTCTCTCCGGACAATTCCCCTCCGAGATGTTGATCCGCGCGCCACTCTCCTCGCGGATCCTCTTAACCGACTCCCCTTTCTTCCCAATGATGCTTCC
```

```
Ref   TACTTCCTTTCCGTGCATAAGAAGCCGAATGGTGAGAGTCACATTTAGTC-CACTTTCAGTCACACCGGCATCCATGGCGAGCGGCGGGC-GG
Seq1 TACTTCCTTTCCGTGCATAAGAAGCCGAATGGTGAGAGTCACATTTAGTC-CACTT-----------GGCATCCCTGCCGCGCGGCGGGCGGG
Seq2 TACTTCCTTTCCGTGCATAAGAAGCCGAATGGTGAGAGTCACATTCAGTCACACC----GTCACACAGGGATACATGGCGAGCGCGTTGCGGG
```

SUM159 ARIH1 sh5 hnRNP E1 CRISPR clone 1 product B (~300 bp)

```
Ref   CGGTACTGTTGGTCATGGAGCTGTTGATATCTTCCTCCAGCTTGTCGATGATCATAGCGAAAGCCTTAAAGATGGCATTGGTGGGGCCGGTCAGAGTGAT
Seq1 CGGTA-----------------------------------------------------------------------------------------AGT---
Seq2 CTG------------------------------CCTCCA-------------------------------------------------------------

Ref   GATTCTCTCCGGACAATTCCCCTCCGAGATGTTGATCCGCGCGCCACTCTCCTCGCGGATCCTCTTAACCGACTCCCCTTTCTTCCCAATGATGCTTCCT
Seq1 ---------------------------------------------------CTCGC--------------------------------------------
Seq2 ----------------------------------------------------------------------------------------------------

Ref   ACTTCCTTTCCGTGCATAAGAAGCCGAATGGTGAGAGTCACATTTAGTCCACTTTCAGTCACACCGGCATCCATGGCGAGCGGCGGGCGGCGTTCGGGG
Ref   -------------------------------------------------------------CAGCGGC---CATGG-------------GCGGTCGCGGG
Seq2 ------------------------------------------------------------ACACGGGCATCGATGGCGATCGGGGGGAGGCGGGCGGGGG

Ref   AGTTGGGCTCGTTA
Seq1 CGTTGTTCTCGTTA
Seq2 ACGTGGGCTCGTTA
```

SUM159 ARIH1 sh5 hnRNP E1 CRISPR clone 16 product A (550 bp)

```
Ref   TCGCGGATCTCTTTGATCTTACACCCGCCTTTCCCAATCAGGGAGCCGCACTGGGTGGCCGGCACCACCAGCCTCAGGGTGACCGGGGGCCTGCTGGCCG
Seq1 TCGCGGATCTCTTTGATCTTACACCCGCCTTTCCCAATCAGGGAGCCGCACTGGGTGGCCGGCACCACCAGCCTCAGGGTGACCGGGGGCCTGCTGGCCG
Seq2 TCGCGGATCTCTTTGATCTTACACCCGCCTTTCCCAATCAGGGAGCCGCACTGGGTGGCCGGCACCACCAGCCTCAGGGTGACCGGGGGCCTGCTGGCGG

Ref   CGGTACTGTTGGTCATGGAGCTGTTGATATCTTCCTCCAGCTTGTCGATGATCATAGCGAAAGCCTTAAAGATGGCATTGGTGGGGCC---GGTCAGAGT
Seq1 CGGTACTGTTGGTCATGGAGCTGTTGATATCTTCCTCCAGCTTGTCGATGATCATAGCGAAAGCCTTAAAGATGGCATTGGTGGGGCC---GGTCAGAGT
Seq2 CGGT------------GGAGCTGGTGATGTATTTGTCCAAATTGTCGACGATCTTGGCGAAAATCTTAAAGAAGGCATTGGAGAGGACATTGGT---AGG

Ref   GATGATTCTCTCCGGACA--ATTCCCCTCCGAGATGTTGATCCGCGCGCCACTCTCCTCGCGGATCCTCTTAACCGACTCCCCTTTCT--TCCCAATGAT
Seq1 GATGATTCTCTCCGGACA--ATTCCCCTCCGAGATGTTGATCCGCGCGCCACTCTCCTCGCGGATCCTCTTAACCGACTCCCCTTTCT--TCCCAATGAT
Seq2 GATGATTCTATC--GATAATATTCTCCTGACAAATGCTGATCCACACGCCGATCTCCTCGCCGATCCTCTTAACGAACTCCCTTTTCTACTCCCCT--AT

Ref   GCTTCCTACTTCCTTTCCGTGCATAAGAAGCCGAATGGTGAGAGTCACATTTAGTC-CACTTTCAGTCACACCGGCATCCATGGCGAGCGGCGGGCGGCG
Seq1 GCTTCCTACTTCCTTTCCGTGCATAAGAAGACGAATGGTGAGAGTCACATTTAGTC-CAC------------CGGCATCCATGGCGAGCGGCGGGCGGCG
Seq2 CTTTCCTACTATGTTTCCTAGTATCTTAACCCGAATGGTAAGACTAACGTTGAGACTCATTTTTACTC-CACCGGCATCCATGGCAAGCGGCGGGCGGCG
```

SUM159 ARIH1 sh5 hnRNP E1 CRISPR clone 16 product B (~300 bp)

```
Ref   GTACTCTCGC-GGATCTCTTTGATCTTACACCCGCCTTTCCCAATCAGGGAGCCGCACTGGGTGGCCGGCACCACCAGCCTCAGGGTGACCGGGGGCCTG
Seq1 GTACTCTCGCGGGATCTCTTTGATCTTACACCCGCCTTTCCCATTCAAGGAGCCGCGATGGGTGGCCGGCACCACCAGCCTCAGGGTGACCGGGGGCCTG
Seq2 GTACAGTCGCGTCATCTCTTTGATCTTACAACCGCCTTTCCCAATCAAGGAGTCGCACTGGGTGGCCGGCACCACCAGCCTCAGGGTGACCGGGGGCCTG

Ref   CTGGCCGCGGTACTGTTGGTCATGGAGCTGTTGATATCTTCCTCCAGCTTGTCGATGATCATAGCGAAAGCCTTAAAGATGGCATTGGTGGGGCCGGTCA
Seq1 ATGGCCGCG----------------ACCTGT---------------------------------------------------------------------
Seq2 CTGGTCGCTGT-----------------------------------------------------------------------------------------

Ref   GAGTGATGATTCTCTCCGGACAATTCCCCTCCGAGATGTTGATCCGCGCGCCACTCTCCTCGCGGATCCTCTTAACCGACTCCCCTTTCTTCCCAATGAT
Seq1 ----------------------------------------------------------------------------------------------------
Seq2 ----------------------------------------------------------------------------------------------------

Ref   GCTTCCTACTTCCTTTCCGTGCATAAGAAGCCGAATGGTGAGAGTCACATTTAGTCCACTTTCAGTCACACCGGCATCCATGGCGAGCGGCGGGCGGCGT
Seq1 ------------------------------------------------------------------CACACCAGCATCCATGGCGAGCGGCGGGCGGCGT
Seq2 --------------------------------------------------------------CAGGCACACCAGCATCCATGGCGAGCGGCGGGCGGCGT

Ref   TCGGGGGAGTTGGGCTCGTTACGGGG
Seq1 TCGGGGGAGTTGGGCTCGTTACGTGG
Seq2 TCGGGGGAGTTGGGCTCGTTACGTGG
```

SUM159 ARIH1 sh5 hnRNP E1 CRISPR clone 20 product A (550 bp)

```
Ref   CGGT-ACTGTTGGTCATG--GAGCTGTTGATATCTTCCTCCAGCTTGTCGATGATCATAGCGAAAGCCTTAAAGATGGCATTGGTGGGGCCGGTCAGAGT
Seq1 CGGT-ACTGTTGGTCATG--GAGCTGTTGATATCTTCCTCCAGCTTGTCGATGATCATAGCGAAAGCCTTAAAGATGGCATTGGTGGGGCCGGTCAGAGT
Seq2 GTGTGACCGGCATCCATGGCGAGCGGAGGG---CGGCGTTCAGCTTGTCTATGATCATAGCGAAAGCCTGAAAGATGGCATTGGTGGGGCCGGTCAGAGT

Ref   GATGATTCTCTCCGGACAATTCCCCTCCGAGATGTTGATCCGCGCGCCACTCTCCTCGCGGATCCTCTTAACCGACTCCCCTTTCTTCCCAATGATGCTT
Seq1 GATGATTCTCTCCGGACAATTCCCCTCCGAGATGTTGATCCGCGCGCCACTCTCCTCGCGGATCCTCTTAACCGACTCCCCTTTCTTCCCAATGATGCTT
Seq2 GATGATTCTCTCCGGACAATTCCCCTCCGAGATGTTGATCCGCGCGCCACTCTCCTCGCGGATCCTCTTAACCGACTCCCCTTTCTTCCCAATGATGCTT

Ref   CCTACTTCCTTTCCGTGCATAAGAAGCCGAATGGTGAGAGTCACATTTAGTCCACTTTCAGTCACACCGGCATCCATGGCG
Seq1 CCTACTTCCTTTCCGTGCATAAGAAGCCGAATGGTGAGAGTCACATTTAGTCCACTTTCAGTCACACCGGCATCCATGGCG
```

```
Seq2 CCTACTTCCTTTCCGTGCATAAGAAGCCGAATGGTGAGAGTCACATT-----------CAGTCACACCGGCATCCATGGCG
```

SUM159 ARIH1 sh5 hnRNP E1 CRISPR clone 20 product B (~300 bp)

```
Ref  TCGCGGATCTCTTTGATCTTACACCCGCCTTTCCCAATCAGGGAGCCGCACTGGGTGGCCGGCACCACCAGCCTCAGGGTGACCGGGGGCCTGCTGGCCG
Seq1 TCGCGGATCTCTTTGATCTTACACCCGCCTTTCCCAATCAAGGAGCCGCACTGGGTGGCCGGCACCACCAGCCTCAGGGTGACCGGGGGCCTGCTGGCCG
Seq2 TCGCGGATCTCTTTGATCTTACAACCGCCTTTCCCATTCAGGGAGCCGCGATGGGTGGCCGGCACCACCAGCCTCAGGGTGACCGGGGGCCTGATGGTCG

Ref  CGGTACTGTTGGTCATGGAGCTGTTGATATCTTCCTCCAGCTTGTCGATGATCATAGCGAAAGCCTTAAAGATGGCATTGGTGGGGCCGGTCAGAGTGAT
Seq1 ---------------------------------------------------------------------------------------------GTGTGA-
Seq2 ----------------------------------------------------------------------------------------------------

Ref  GATTCTCTCCGGACAATTCCCCTCCGAGATGTTGATCCGCGCGCCACTCTCCTCGCGGATCCTCTTAACCGACTCCCCTTTCTTCCCAATGATGCTTCCT
Seq1 ----------------------------------------------------------------------------------------------------
Seq2 ----------------------------------------------------------------------------------------------------

Ref  ACTTCCTTTCCGTGCATAAGAAGCCGAATGGTGAGAGTCACATTTAGTCCACTTTCAGTCACACCGGCATCCATGGCGAGCGGCGGGCGGCGTTCGGGG
Seq1 ---------------------------------------------------------------CCGGCATCCATGGCGAGCGGCGGGCGGCGTTCGGGG
Seq2 ------------------------------GTGCGA---------------------------CCGGAATTAATGGCGAGCGGCGGGCGGCGTTCGGGG
```

SUM159 ARIH1 sh5 hnRNP E1 CRISPR clone 54 product (550 bp)

```
Ref  TCGCGGATCTCTTTGATCTTACACCCGCCTTTCCCAATCAGGGAGCCGCACTGGGTGGCCGGCACCACCAGCCTCAGGGTGACCGGGGGCCTGCTGGCCG
Seq1 TCGCGGATCTCTTTGATCTTACACCCGCCTTTCCCAATCAGGGAGCCGCACTGGGTGGCCGGCACCACCAGCCTCAGGGTGACCGGGGGCCTGCTGGCCG
Seq2 TCGCGGATCTCTTTGATCTTACACCCGGATCTTCCTATCAGGGGGCCGTGCTGATTGGCAAGCACTTCCAGACTGAAGGTGAGGGGGGGGCCGCAGGGAT

Ref  CGGTACTGT-TGGTCATGGAGCTGTTGATATCTT-------CCTCCA-GCTTGTCGATGATCATAGCGAAAGCCTTAA-AGA-TGGCATTGGTGGGGCCG
Seq1 CGGTACTGT-TGGTCATGGAGCTGTTGATATCTT-------CCTCCA-GCTTGTCGATTATCATAGCGAAAGCCTTAA-AGA-TGGCATTGGTGGGGCCG
Seq2 CAGT-CTCTCCGGACAATTACCTGCTGATATGTTGATCCGCGCTCCACTCTCCTCTCAGATCCT---------CTTAACCGACTCCCCTTTCTTCGGCCG

Ref  GTCAGAGTGATGATTCTCTCCGGACAATTCCCCTCCGAGATGTTGA---TCCGCGCGCCACTCTCCTCGCGGATCCTCTTAACCGACTCCCCTTTCTTCC
Seq1 GTCAGAGTGATGATTCTCTCCGGACAATTCCCCTCCGAGATGTTGA---TCCGCGCGCCACTCTCCTCGCGGATCCTCTTAACCGACTCCCCTTTCTTCC
Seq2 G-CAGAATGATGATTCTCTTCCGACCATTCCCCTCCCAAAAGT-GAGAGTCCCCGC--CACTCCCCTTTCCGATCCTCTTAACCCACTCCCCATTCGTCG

Ref  CAAT-GATGCTTCCTACTTCCTTTCCGTGCATAAGAAGCCGAATGGTGAGAGTCACATTTAGTCCAC-TTTCAGTCACACCGGCATCCATGGCGAGCGGC
Seq1 CAAT-GATGCTTCCTACTTCCTTTCCGTGCATAAGAAGCCGAATGGTGAGAGTCACATTTAGTCCAC-TTTCAGTCACACCGGCATCCATGGCGAGCGGC
Seq2 GAATGGATGCT-CCGACTTTCTTTTCGTGGATTAGAAAGCAAAAGGTGAGAAGCACATTTAATCCACTTTTCATTCCCCCCGGTTTCATTGGAAAGGGGG
```

SUM159 ARIH1 sh5 hnRNP E1 CRISPR clone 11 product (550 bp)

```
Ref  TCGCGGATCTCTTTGATCTTACACCCGCCTTTCCCAATCAGGGAGCCGCACTGGGTGGCCGGCACCACCAGCCTCAGGGTGACCGGGGGCCTGCTGGCCG
Seq1 TCGCGGATCTCTTTGATCTTACACCCGCCTTTCCCAATCAGGGAGCCGCACTGGGTGGCCGGCACCACCAGCCTCAGGGTGACCGGGGGCCTGCTGGCCG
Seq2 TCGCGGATCTCTTTGATCTTACACCCGCCTTTCCCAATCAGGGAGCCGCACTGGGTGGCCGGCACCACCAGCCTCAGGGTGACCGGGGGCCTGCTGGCCG

Ref  CGGTACTGTTGGTCATGGAGCTGTTGATATCTTCCTCCAGCTTGTCGATGATCATAGCGAAAGCCTTAAAGATGGCATTGGTGGGGCCGGTCAGAGTGAT
Seq1 CGGTACTGTTGGTCATGGAGCTGTTGATATCTTCCTCCAGCTTGTCGATGATCATAGCGAAAGCCTTAAAGATGGCATTGGTGGGGCCGGTCAGAGTGAT
Seq2 CGGTACTGGTGGGCATGGAGCTGTTGATATCTTCCTCCAGCTTGGCGATGATCATAACGAAAGCCTTAAAGATGGGATTGGTGGGGCCGGGCAGAATGAT

Ref  GATTCTCTCCGGACAATTCCCCTCCGAGATGTTGATCCGCGCGCCACTCTCCTCGCGGATCCTCTTAACCGACTCCCCTTTCTTCCCAATGATGCTTCCT
Seq1 GATTCTCTCCGGACAATTCCCCTCCGAGATGTTGATCCGCGCGCCACTCTCCTCGCGGATCCTCTTAACCGACTCCCCTTTCTTCCCAATGATGCTTCCT
Seq2 GAATCTCTCCGGACAATTCCCCTCCGAGATGGTGATCCGCGCGCCACTCTCCTCGCGGATCCTCTTAACCGACTCCCCTTTCTTCCCAATGATGCTTCCT

Ref  ACTTCCTTTCCGTGCATAAGAAGCCGAATGGTGAGAGTCACATTTAGTCCACTTTCAGTCACACCGGCATCCATGGCG
Seq1 ACTTCCTTTCCGTGCATAAGAAGCCGAATGGTGAGAGTCACATTTAGTCCACTTTCAGTCACACCGGCATCCATGGCG
Seq2 ACTTCCTTTCCGTGCATAAGAAACCGAATGGTGAGAATCACATTTAATCCACTT-CAGTCACACCGGCATCCATGGGG
```

SUM159 ARIH1 sh5 hnRNP E1 CRISPR clone 37 product (550 bp)

```
Ref  TCGCGGATCTCTTTGATCTTACACCCGCCTTTCCCAATCAGGGAGCCGCACTGGGTGGCCGGCACCACCAGCCTCAGGGTGACCGGGGGCCTGCTGGCCG
Seq1 TCGCGGATCTCTTTGATCTTACACCCGCCTTTCCCAATCAGGGAGCCGCACTGGGTGGCCGGCACCACCAGCCTCAGGGTGACCGGGGGCCTGCTGGCCG
Seq2 TCGCGGATCTCTTTGATCTTACACCCGCCTTTCCCAATCAGGGAGCCGCACTGGGTGGCCGGCACCACCAGCCTCAGGGTGACCGGGGGCCTGCTGGCCG

Ref  CGGTACTGTTGGTCATGGAGCTGTTGATATCTTCCTCCAGCTTGTCGATGATCATAGCGAAAGCCTTAAAGATGGCATTGGTGGGGCCGGTCAGAGTGAT
Seq1 CGGTACTGTTGGTCATGGAGCTGTTGATATCTTCCTCCAGCTTGTCGATGATCATAGCGAAAGCCTTAAAGATGGCATTGGTGGGGCCGGTCAGAGTGAT
Seq2 CGGTACTGTTGGTCATGGAGCTGTTGATATCTTCCTCCAGCTTGTCGATGATCATAGCGAAAGCCTTAAAGATGGCATTGGTGGGGCCGGTCAGAGTGAT

Ref  GATTCTCTCCGGACAATTCCCCTCCGAGATGTTGATCCGCGCGCCACTCTCCTCGCGGATCCTCTTAACCGACTCCCCTTTCTTCCCAATGATGCTTCCT
Seq1 GATTCTCTCCGGACAATTCCCCTCCGAGATGTTGATCCGCGCGCCACTCTCCTCGCGGATCCTCTTAACCGACTCCCCTTTCTTCCCAATGATGCTTCCT
Seq2 GATTCTCTCCGGACAATTCCCCTCCGAGATGTTGATCCGCGCGCCACTCTCCTCGCGGATCCTCTTAACCGACTCCCCTTTCTTCCCAATGATGCTTCCT

Ref  ACTTCCTTTCCGTGCATAAGAAGCCGAATGGTGAGAGTCACATTTAGTCCACTTTCAGTCACACCGGCATCCATGGCG
Seq1 ACTTCCTTTCCGTGCATAAGAAGCCGAATGGTGAGAGTCACATTTAGTCCACTTTCAGTCACACCGGCATCCATGGCG
Seq2 ACTTCCTTTCCGTGCATAAGAAGCCGAATGGTGAGAGTCACATTTAGTCCACTTT----------GGCATCCATGGGG
```
